# Let's talk about faces: Identifying faces from verbal descriptions

**DOI:** 10.1111/bjop.12610

**Published:** 2022-11-04

**Authors:** Rebecca Tyler, Alice Towler, Richard I. Kemp, David White

**Affiliations:** ^1^ School of Psychology University of New South Wales Sydney New South Wales Australia

**Keywords:** discourse, face perception, face recognition, facial descriptions, individual differences, super‐recognizers, verbal ability, verbal communication

## Abstract

Face descriptions inform real‐world identification decisions, for example when eyewitnesses describe criminal perpetrators. However, it is unclear how effective face descriptions are for identification. Here, we examined the accuracy of face identification from verbal descriptions, and how individual differences in face perception relate to producing and using descriptions for identification. In Study 1, participants completed a face communication task in pairs. Each participant saw a single face, and via verbal communication only, the pair decided if they were viewing the same person or different people. Dyads achieved 72% accuracy, compared to 81% when participants completed the task individually by matching face pairs side‐by‐side. Performance on the face communication and perceptual matching tasks were uncorrelated, perhaps due to low measurement reliability of the face communication task. In subsequent studies, we examined the abilities of face ‘describers’ (Study 2) and ‘identifiers’ separately (Study 3). We found that ‘super‐recognizers’ – people with extremely high perceptual face identification abilities – outperformed controls in both studies. Overall, these results show that people can successfully describe faces for identification. Preliminary evidence suggests that this ability – and the ability use facial descriptions for identification – has some association with perceptual face identification skill.

## INTRODUCTION

Describing faces and identifying faces from descriptions are important tasks in social interactions and applied settings. We may describe someone's facial appearance to a friend when trying to ascertain if we are talking about the same person (e.g., ‘she has green eyes and a big smile’). Face descriptions can also be important in criminal investigations. For example, a witness may be asked to describe an offender to police to construct a likeness using a composite system or sketch, or a description may be transmitted to officers in the field via radio.

Despite being an important and relatively common task, little is known about how people describe faces for identification purposes. There have been some rare attempts to examine free descriptions of faces in studies regarding social attributions made to faces (e.g., Oosterhof & Todorov, 2008), but studies on the description of face identity have mostly been concerned with the content of facial descriptions made by eyewitnesses. Overwhelmingly, eyewitnesses report very few details about offenders' faces and their descriptions of facial information tend to be highly error‐prone – indicating facial descriptions are of poor quality for *recognition* purposes (Sporer, 1992; Van Koppen & Lochun, 1997). However, the sparseness and inaccuracies in eyewitness facial descriptions may be due to memory constraints, as the nature of the task involves a delay between initial viewing of the face, generating a description, and completing a subsequent face recognition test (Lindsay et al., 2011). The eyewitness literature is therefore limited in offering insights into whether the process of generating facial descriptions for identification, independent of memory demands, is error‐prone in and of itself. In addition, research in the domain of eyewitness recognition does not address how useful face descriptions are in circumstances when the person making the identification is a different person to the initial describer (Meissner et al., 2007). We therefore know little about how effectively individuals can communicate facial information to others.

In contrast, there is an abundance of research on people's ability to identify faces from perceptual information where there is no requirement to describe the face. Unfamiliar face‐matching decisions, for example when matching a photograph of a suspect to CCTV footage, are highly error‐prone, even when both images are available for perceptual comparison (Bruce et al., 2001; Burton & Jenkins, 2011; Hancock et al., 2000). The error rate in face identification decisions observed in purely perceptual conditions constrains the upper limit of accuracy we could expect to see in situations that involve both perceptual and verbal demands.

As might be expected given the relatively poor performance in tests of perceptual face identification, the few studies that have examined people's ability to identify faces from descriptions suggest this is a very difficult task. In one study, ‘identifiers’ were given facial descriptions generated by participants in a previous study and asked to identify which face, out of five choices, the description pertained to (Fallshore & Schooler, 1995). Relying solely on this facial description, identifier participants selected the correct face at accuracy levels barely above chance (27%). More recently, the utility of face descriptions for identification purposes was investigated in a live matching context (Kramer & Gous, 2020). Here, ‘describers’ generated descriptions of faces in real‐time to ‘identifiers’, who used the description to identify the relevant person from a 10‐person line‐up. When matching a facial description to a different image than that viewed by the describer, identifiers again achieved barely above chance accuracy (23%). These studies offer preliminary evidence that individuals can relay the identity of a face via description, albeit with strikingly low levels of accuracy.

However, all existing studies in the domain of face communication have constrained the exchange of information between individuals to be unidirectional, from describer to identifier (Fallshore & Schooler, 1995; Kramer & Gous, 2020). This prevents reciprocal dialogue between the describer and identifier, for example where an identifier might seek clarification about aspects of the description which were unclear or ambiguous. This is a key limitation in our understanding of face communication because in the real world many face identification decisions involve reciprocal verbal communication in real‐time (e.g., when police radio a suspect description to another officer in the field, who is tasked with apprehending the suspect based on the description).

Although the nature of face communication ability is not well understood, based on other domains, there are reasons to suspect that those with greater perceptual face expertise will also exhibit semantic expertise when describing faces. For example, recent research has demonstrated that individuals who are literate have better object recognition abilities, including for faces, than those who are illiterate, and this effect appears to be driven specifically by learning to read (Van Paridon et al., 2021). These results suggest that the ability to comprehend and express oneself through written language assists with fine‐tuning object recognition skills, although it is unclear to what extent domain‐specific literacy for objects of recognition contributes to these effects. Other evidence for a relationship between perceptual and verbal expertise is found in wine tasting, where through training, individuals can enhance both their verbal/conceptual knowledge about wine as well as their olfactory detection thresholds and discrimination (Block & Beckett, 1990; Spence & Wang, 2019). Wine experts are also more accurate at matching wines to written descriptions than novices (Hughson & Boakes, 2009; Solomon, 1990). This evidence suggests that those with greater perceptual expertise also tend to exhibit semantic expertise within that same domain. However, wine descriptions are often not literal and instead rely on analogy (e.g., flamboyant, toasty, velvety), while face descriptions can be literal (e.g., ‘blue eyes’). Critically, the goals of subjective (e.g., analogy‐based) and objective (fact‐based) description are very different – the former is focused on creating an impression or vision, while the latter is focused on accuracy (Connelly, 2012). Thus, although tasting and describing wines are skills that appear to be closely related, it is not clear whether our perceptual expertise with faces is so closely tied to verbal communication.

Here we test how individual differences in perceptual ability with faces is related to the ability to describe and interpret verbal descriptions of faces. In recent years, it has become clear that there are large differences in people's ability to identify faces. While some people struggle to recognize their closest friends and relatives (prosopagnosia; Duchaine & Nakayama, 2006), others can easily recognize the most trivial of acquaintances even years later (super‐recognizers; Russell et al., 2009). These groups appear to represent the ends of a distribution of ability that varies dimensionally in the population, and this variation is known to be heritable, stable over time and largely unaffected by training/experience (Balsdon et al., 2018; DeGutis et al., 2014; Towler et al., 2019, 2020; Wilmer et al., 2010). However, the cognitive parameters of individual differences in face identification and the extent to which they generalize beyond the primary visual system have been little studied.

Whether individual differences in face identification ability extend to verbal abilities in describing faces is not known, however verbal communication does appear to be associated with identification accuracy in some circumstances. For example, communication between individuals working together to make identity decisions has been found to enhance performance in perceptual face‐matching (Dowsett & Burton, 2015). However, benefits to the accuracy of joint decisions were only conferred by people with high levels of face‐matching ability to those with lower ability. This finding suggests that individuals with strong abilities in matching faces can communicate the basis for their decisions effectively and raises the possibility that they are also better able to describe faces. However, the content of participant discussions leading to this communication benefit is not well understood (Ritchie et al., 2022). Additionally, facial forensic examiners are known to outperform standard participant groups in face identification ability, and unlike other high‐performers in face identification (i.e., super‐recognizers), they are required to support face identification decisions with verbal justifications and specialized face vocabulary (White et al., 2015). However, the aetiology of forensic examiners perceptual expertise and its relationship to their verbal expertise for faces is not well understood. Consequently, understanding the relationship between individual differences in perceptual and verbal expertise for faces is important for theoretical and practical reasons.

In three studies, we measured the accuracy of identification decisions based on facial descriptions. We aimed to improve understanding of the factors influencing this accuracy; in particular, how description generation and comprehension accuracy are associated with an individual's perceptual face identification abilities. In our first study, dyads of participants communicated about faces in real‐time to make perceptual matching decisions, where each participant viewed one image of the image pair. This allowed us to investigate people's ability to identify faces when using natural interactive dialogue. However, this approach did not enable us to examine the separate contributions of face ‘describers’ (i.e., generating descriptions) and face ‘identifiers’ (i.e., identifying based on descriptions). In two subsequent studies, we therefore investigated the contribution of individual differences in perceptual ability for faces towards verbal‐based identification accuracy in face ‘describers’ (Study 2) and ‘identifiers’ (Study 2 & 3) separately.

## STUDY 1

In Study 1, we examined three initial questions. First, we measured the accuracy with which dyads of participants could make face‐matching decisions by utilizing natural two‐way conversation to exchange verbal information about faces. Dyad performance was compared to individual performance on the same task in standard perceptual discrimination conditions.

Second, we examined whether individual differences in perceptual matching ability were associated with the accuracy of dyads in verbally based identification decisions by measuring the correlation across perceptual and verbal versions of the task, and an existing perceptual test with strong psychometric properties. Third, we examined the content of participants' descriptions of facial information to capture the way that people describe faces for the purpose of identification and to test whether certain types of description are associated with higher accuracy.

### Method

#### Participants

A total of 102 first‐year University of New South Wales (UNSW) Psychology students (*M*_age_ = 19.67 years, 68.63% female, 63.73% Caucasian) participated in the study in exchange for course credit. We advertised two openings per timeslot, so that participants were automatically paired with another person in their timeslot. All participants were Native English speakers and had normal or corrected‐to‐normal vision. We required participants in all three studies reported here to speak English as their first language because evidence suggests non‐native speakers are more likely to make linguistic errors, particularly syntax‐related, and have different interpretations of colloquial speech patterns (e.g., idioms, slang) when communicating in their non‐native language (Mäntylä, 2004; Marina & Snuviškiene, 2005). All studies reported here received ethics approval from UNSW.

#### Materials

##### Glasgow Face‐Matching Test (GFMT)

**FIGURE 1:**
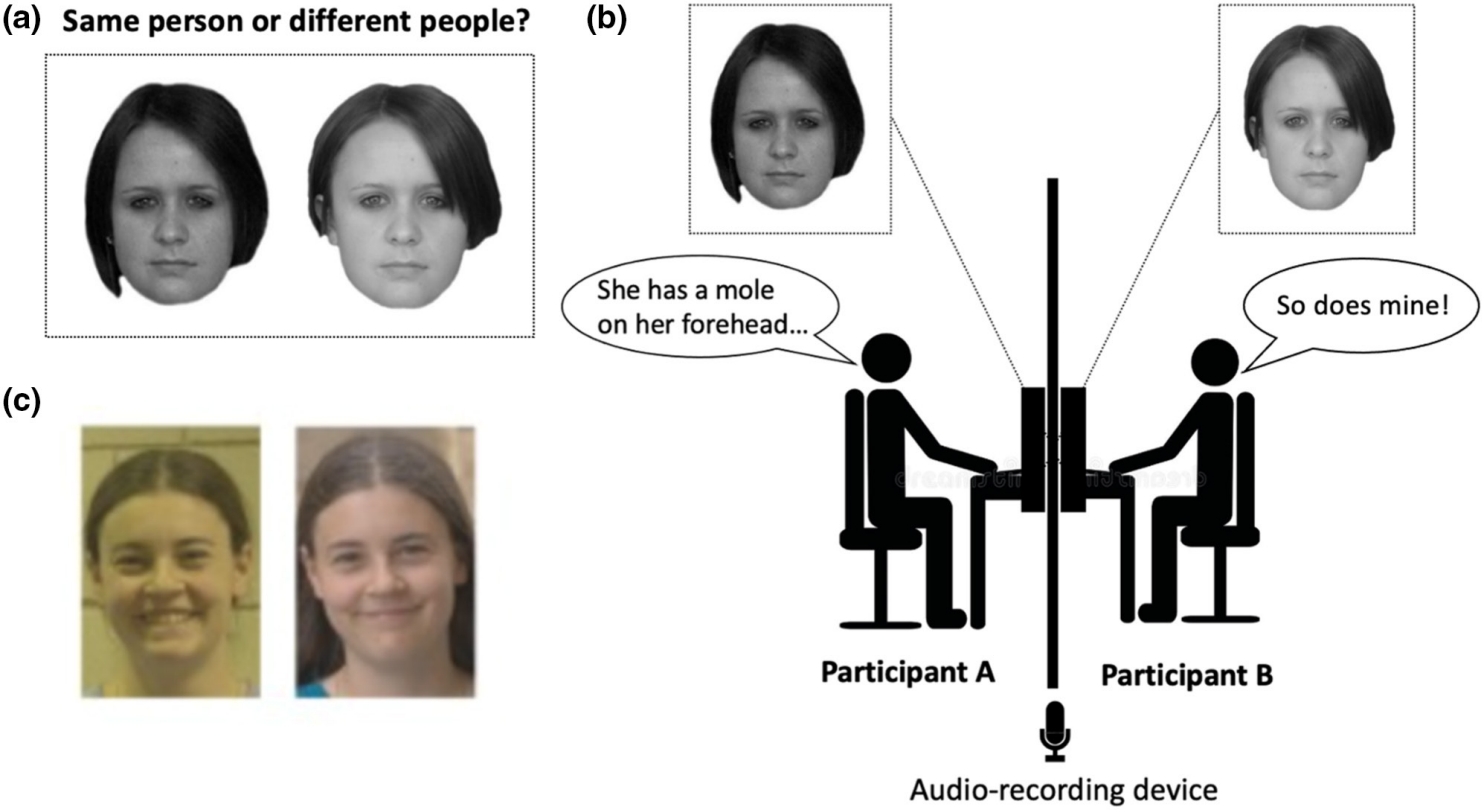
(a) Perceptual GFMT: example trial. In this example, the two images are of the same person. (b) Verbal GFMT: visual representation of the study procedure. Participant A and Participant B were seated opposite each other, separated by a fabric screen, so that they could hear but not see each other. On their respective computer screens, each participant saw an image of a face. In this example, the two images are of the same person. (c) EFCT: example trial. In this example, the two images are of the same person

The GFMT (Burton et al., 2010) is a standardized measure of face‐matching ability and consists of 40 pairs of studio quality black and white photos of Caucasian faces (male and female), in front‐on view. Based on prior testing (Towler et al., 2014), the standard 40‐item version of the GFMT was sub‐divided into two, 20‐item sub‐tests of equal difficulty, each with 10 matching pairs (two different images of the same person) and 10 non‐matching pairs (two images of different people). For each dyad of participants, one of the sub‐tests was randomly selected to be used as a measure of face‐matching ability (‘Perceptual GFMT’; see Figure 1a), and the other was used to measure how well participants can communicate about faces to make a joint identification decision (‘Verbal GFMT’; see Figure 1b). The order of items in both versions was pseudo‐randomized to minimize order effects.

The Perceptual GFMT was administered individually to each participant in the same format as the original GFMT. On each trial, each participant viewed two facial images simultaneously and decided whether the images were of the same person or two different people. For the Verbal GFMT, each member of the dyad was shown only *one* of the two images from the trial of the GFMT. Participants could not see their partner's image. Through discussion only, the dyad sought to determine whether the two images were of the same person or two different people, and one of the participants recorded their collective response.

##### Expertise in facial comparison test (EFCT)

The EFCT (White et al., 2015) is a simultaneous face‐matching test that was designed to mimic the type of task forensic face examiners perform in the course of their work. The EFCT was used to obtain an additional measure of face identification ability. Two versions of the EFCT, known to be equivalent in difficulty based on the original test development, were used as a pre‐test and post‐test (i.e., before and after the Verbal GFMT) of individuals' face‐matching accuracy. We included a pre‐ and post‐test of face identification ability as prior research observed an improvement in performance when participants collaborated on face identity decisions in pairs, but only for individuals who were poor at a baseline test of perceptual matching (Dowsett & Burton, 2015). We therefore wanted to examine if the experience of having to describe faces to each other would be sufficient to elicit improvement in a subsequent perceptual matching task.

In each EFCT version, across 84 matching trials, participants decided whether two simultaneously presented images depicted the same person or different people, and both images remained on screen until a response was made (see Figure 1c). Accuracy was measured using area under the receiver operating characteristic curve (AUC). The order of use of the two versions of the EFCT was kept constant within participant dyads (i.e., Participant A and Participant B completed the same versions as each other at pre‐test and at post‐test) but counterbalanced between dyads.

#### Procedure

Participant A and Participant B were each seated at a computer with a screen separating them to prevent non‐verbal communication. Participants first worked independently at their own computers to complete the EFCT pre‐test and the Perceptual GFMT. Once both participants had completed the Perceptual GFMT, the experimenter provided instructions for the Verbal GFMT. Participants were told to read each trial number aloud to ensure they were comparing the correct images, and then to take it in turns to describe their images to each other so they could reach a joint identification decision. They were told that their conversations would be audio‐recorded, to allow for coding of their verbal responses. After completing the 20 trials of the Verbal GFMT, participants worked independently to complete the post‐test EFCT.

Most participants took 45–60 min to complete all tasks. Three dyads did not complete the EFCT post‐test due to time constraints (a maximum of 60 min was allowed for the study). Due to technical difficulties, one dyad was not recorded while undertaking the Verbal GFMT. These four dyads were included in analyses, excluding those that their missing data affected. All statistical analyses reported here were completed in IBM SPSS Statistics Software.

### Results

#### Perceptual and verbal face identification accuracy

**FIGURE 2:**
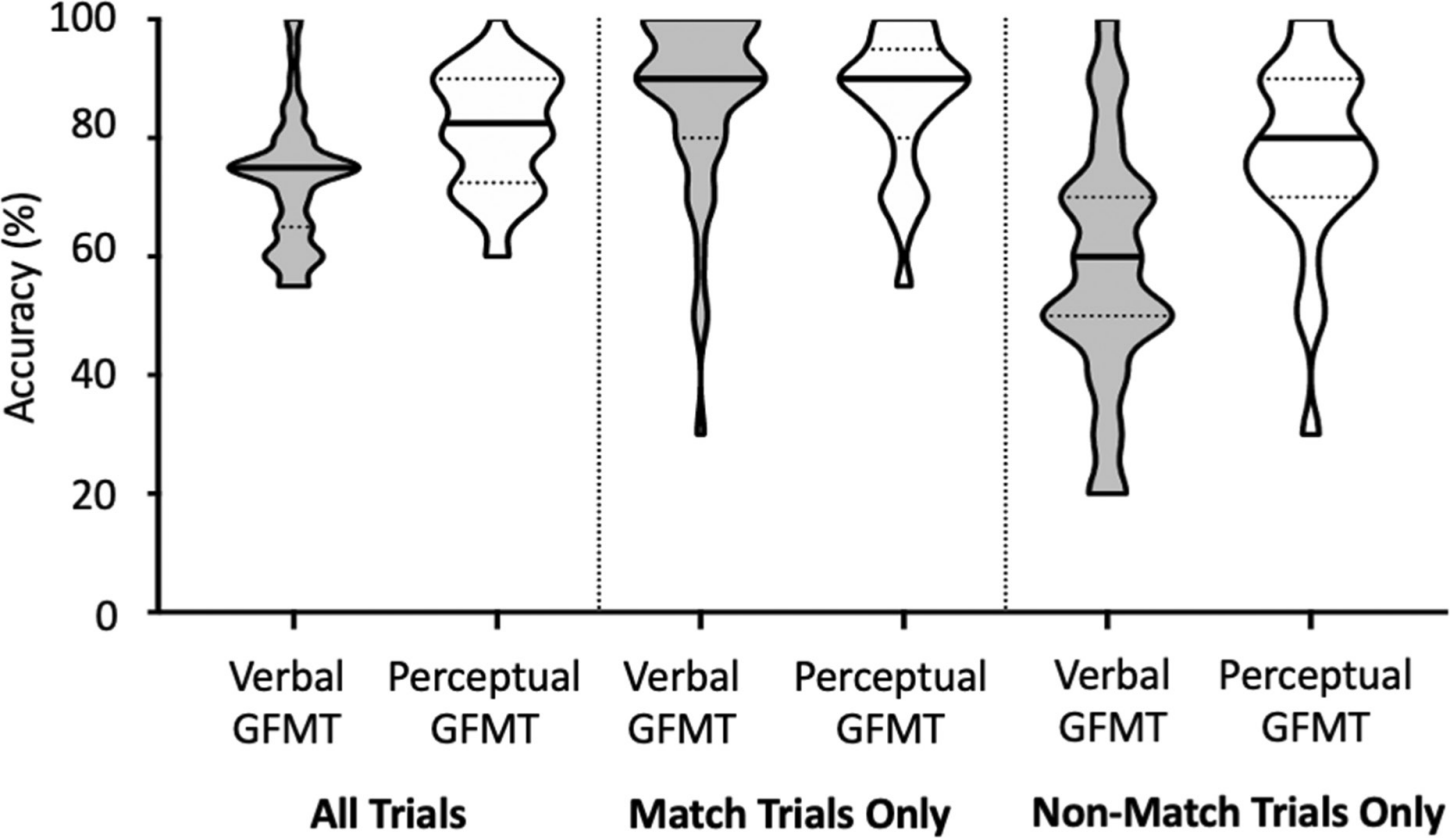
Dyads' performance on the verbal GFMT and perceptual GFMT for all trials, match trials only, and non‐match trials only. The solid line on each violin plot represents the median accuracy. The area between the dotted lines on each violent plot represents the interquartile range

As the first step in our analysis, we wanted to examine the accuracy with which participants can make face‐matching decisions based on collaborative verbal communication, and to compare this level of performance to that achieved in the standard GFMT when participants could visually inspect and compare the pair of images. Accuracy on the Verbal GFMT and Perceptual GFMT are shown in Figure 2. Participants' overall accuracy on the Perceptual GFMT and the tendency for higher accuracy in match trials of this test are consistent with published normative data (Burton et al., 2010).

Mean accuracy on the Verbal GFMT (*M* = 72.5%, *SD* = 10.9) was substantially above chance, but significantly lower than mean accuracy on the Perceptual GFMT (*M* = 81.3%, *SD* = 9.8), *t*(50) = −4.72, *p* < .001, Cohen's *d* = .85 [95% CI: −4.728, −12.872]. Analysis of the match and non‐match trials separately revealed no significant differences in accuracy between the Verbal and Perceptual GFMTs for match trials (*M*_VerbalGFMT_ = 87.1%, *M*_PerceptualGFMT_ = 85.7%, *t*(50) = −0.06, *p* = .950, Cohen's *d* = .09 [95% CI: −4.734, 7.534]). In contrast, for non‐match trials, we observed lower accuracy on the Verbal GFMT than the Perceptual GFMT (*M*_VerbalGFMT_ = 57.8%, *M*_PerceptualGFMT_ = 77.0%, *t*(50) = 4.58, *p* < .001, Cohen's *d* = .98 [95% CI: 11.474, 26.926]).

To examine if communicating about face identity with another person impacted accuracy on subsequent perceptual judgements, we compared performance on the EFCT subtests taken before and after the verbal GFMT. Because prior work has shown that interventions can differentially affect high‐ and low‐performing participants (Dowsett & Burton, 2015; Towler et al., 2021; White et al., 2015), we examined here whether the difference in performance on the EFCTs completed before and after the Verbal GFMT was affected by participants' face identification ability as measured by an independent test (i.e., the Perceptual GFMT). Participants were separated into low and high performers on the Perceptual GFMT based on a median split. A two‐way ANOVA with factors Perceptual GFMT Score (low, high) and EFCT Test Phase (pre, post) showed a significant main effect of EFCT Test Phase, *F*(1, 81) = 4.08, *p* = .047, *η* ^2^ = .008. This reflected a fall in accuracy for the EFCT completed after the Verbal GFMT, with performance on the EFCT at pre‐test (*M*_AUC_ = .83, *SD* = .06) significantly higher than performance at post‐test (*M*_AUC_ = .81, *SD* = .05). However, the interaction was not significant, *F*(1, 81) = .94, *p* = .335, *η* ^2^ = .002, suggesting both high and low performers experienced an accuracy decrement on the EFCT completed after the Verbal GFMT. Thus, we found no evidence that verbally communicating about faces leads to an improvement in perceptual face‐matching performance, and some evidence that it impaired accuracy.

#### Individual differences in perceptual and verbal face identification

**FIGURE 3:**
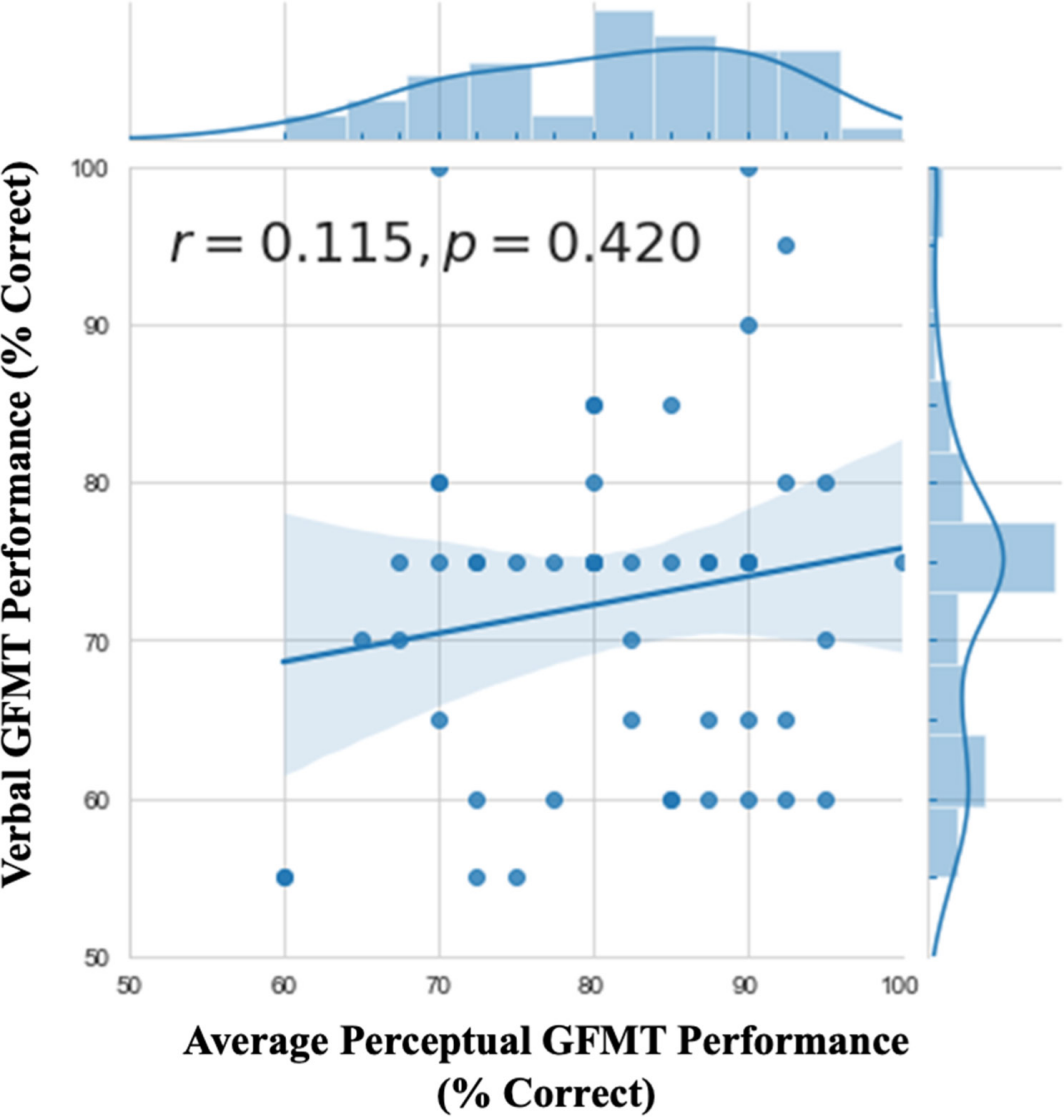
Performance of dyads on the verbal GFMT as a function of their mean accuracy on the Perceptual GFMT

We next asked whether individual participants' perceptual matching ability was predictive of the Verbal GFMT score achieved by the dyad that the participant contributed to. All correlational analyses are reported using Spearman's rho (*r*_s_). As shown in Figure 3, we found large variation in performance for both perceptual and verbal versions of the GFMT. Despite these large differences, we found no significant relationship between performance on the Verbal GFMT and mean performance of the pair on the Perceptual GFMT, *r*_*s*_(49) = .115, *p* = .420, or on the EFCT, *r*_*s*_(49) = .108, *p* = .450. We found a similar pattern of results when examining the relationship between dyads' Verbal GFMT scores and Perceptual GFMT scores for the best and worst‐performing dyad members separately (see Supporting Information).

#### Content of facial descriptions for identification purposes

**FIGURE 4:**
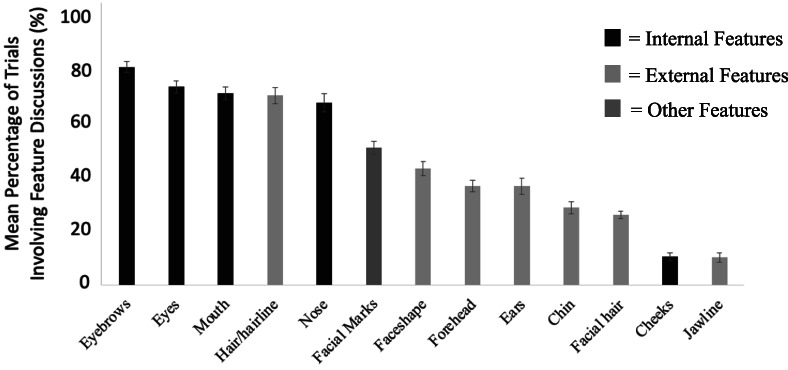
Mean number of trials individual facial features were discussed. Error bars plotted using standard error of the mean

We also examined the content of participants' facial descriptions. Independent raters listened to Verbal GFMT audio recordings in order to code which facial features were mentioned in conversation. For each Verbal GFMT trial completed by each dyad, raters made a binary judgement as to whether facial features were mentioned or not (features coded for were: eyebrows, eyes, mouth, hair/hairline, nose, facial marks, face shape, forehead, ears, chin, facial hair, cheeks, and jawline). Initial inter‐rater agreement (calculated using the percentage of absolute agreement; Altman, 1990; Chaturvedi & Shweta, 2015) for all recordings was above 85%, and all disagreements were resolved by an independent third rater. The results are presented in Figure 4.

For each trial, participants discussed an average of 6.18 different facial features (*SD* = 1.06). Of these mentions, 50% were internal features (eyes, eyebrows, nose, mouth, cheeks), 42% were external features (forehead, chin, jawline, face shape, facial hair, hair, ears), and 8% were features that could not clearly be classified as internal or external (e.g., facial marks such as scars and blemishes). Participants discussed internal features (*M* = 61.9%, *SD* = 16.8) on significantly more trials than they did external features (*M* = 36.7%, *SD* = 16.5), *t*(49) = 5.52, *p* < .001, Cohen's *d* = 1.51 [95% CI: 18.591, 31.809]. The eyebrows were the most discussed internal feature (*M*_%TrialsMentioned_ = 82.4, *SD* = 1.50), and hair was the most discussed external feature (*M*_%TrialsMentioned_ = 71.6, *SD* = 2.15).

In addition to discussion of discrete facial features, most participants (*N* = 36, 72%) used holistic descriptors (e.g., comments about attractiveness, celebrity likeness, personality, vocation). Participants who included holistic descriptors in their discussions mentioned them on 17.36% trials, on average. There was no statistically significant difference in accuracy for the Verbal GFMT overall, for match trials only, or for mismatch trials only, between participants who used holistic descriptors compared to those who did not (Overall Accuracy: *t*(49) = 0.11, *p* = .913, Cohen's *d* = .04 [95% CI: −3.395, 4.195]; Match Accuracy Only: *t*(49) = 0.40, *p* = .689, Cohen's *d* = .13 [95% CI: −3.697, 7.497]; Mismatch Accuracy Only: *t*(49) = −0.66, *p* = .514, Cohen's *d* = .22 [95% CI: −3.622, 12.822]).

We also wanted to understand which quantitative or qualitative aspects of the process of communicating about faces were driving the large variation in Verbal GFMT performance (see Figure 3). We examined factors which might be associated with face communication effectiveness as measured by the Verbal GFMT score, including time spent on the Verbal GFMT, number of features discussed, use of holistic descriptors, and number of trials where individual facial features were discussed. Although the number of trials involving descriptions of facial marks was moderately inversely related to overall accuracy (*r*_*s*_[48] = −.367, *p* = .009) and the number of trials involving descriptions of ears was moderately inversely correlated to match trial accuracy (*r*_*s*_[48] = −.308, *p* = .030), these correlations were not significant after Bonferroni correction. No other factor was significant in predicting Verbal GFMT performance (see Supporting Information).

### Discussion

Results of Study 1 suggest that people can communicate about the appearance of a face to another person reasonably effectively, enabling 72% accuracy on our newly developed Verbal GFMT. This represents a 10% reduction in accuracy when compared to individual participants viewing both images on a computer screen simultaneously. Interestingly, this accuracy reduction was driven by poorer Verbal GFMT performance in non‐match pairs only, pointing to a strong bias to make ‘match’ responses when people describe faces to one another. The causes of this are unclear, but may reflect confirmation bias (Nickerson, 1998) – that is, when one participant described the appearance of a feature on their image, their partner was more likely to agree that their image also contained a feature of similar appearance.

We additionally found evidence that verbally communicating about faces led to a modest, yet statistically significant, reduction in subsequent perceptual face‐matching performance. Although it is possible that this is simply task fatigue, the reduction may suggest that verbalization of face information leads to an impairment in subsequent simultaneous discrimination of faces. Of note, some studies of eyewitness memory have found that describing faces from memory leads to subsequent poorer recognition of those same faces, and this effect is larger for individuals who have higher perceptual expertise for faces (‘verbal overshadowing effect’; Ryan & Schooler, 1998). The question of whether the task of describing faces causes an impairment in subsequent simultaneous discrimination of different faces, and whether this is related to an individual's perceptual expertise for faces, requires further investigation.

We also observed large variation in face communication performance, which was not explained by perceptual face identification ability, the discrete facial features discussed by participants, or overall comments about facial appearance. However, it was not clear whether the variation in Verbal GFMT performance was due to individual differences per se, or test unreliability (White & Burton, 2022). We conducted post‐hoc analyses to examine the internal reliability of measures used to assess perceptual face identification ability and verbal face skill and found both tests were below accepted psychometric thresholds (Perceptual GFMT: *α* = .621, Verbal GFMT: *α* = .316). Consequently, based on the data from Study 1 alone, we cannot rule out an association between verbal and perceptual face identification accuracy, and so we follow up this question in Studies 2 and 3.

While Study 1 examined a relatively naturalistic and interactive style of communication about faces, this design did not allow us to examine the independent contributions of the people generating descriptions (henceforth ‘describers’) and the people that interpreted descriptions (henceforth ‘identifiers’). Therefore, in Studies 2 and 3 we isolated these contributions. Additionally, given the reliability concerns identified in Study 1, in Studies 2 and 3 we also make use of an extreme‐groups design, comparing super‐recognizers – individuals with exceptional face identification skill – to ‘control’ individuals with normative levels of face identification skill. An extreme groups design provided more statistical power to detect an effect of face identification skill on face communication ability, if one exists (Feldt, 1961). This is consistent with the approach used in many other studies of individual differences in face perception (see White & Burton, 2022 for a review) and with evidence that super‐recognizers superior ability exists on a continuum of ability with typical viewers (Dunn et al., 2022; Noyes et al., 2017).

## STUDY 2

In Study 2, we examined whether the functional quality of the face descriptions generated by ‘describers’ was predicted by their face identification ability. Although we did not find a relationship between face communication accuracy and face identification ability in Study 1, this may have been due to the relatively poor psychometric properties of measures of verbal and perceptual ability, and by the fact that contributions of the individuals sending and receiving verbal information were too heavily intertwined.

To provide a more powerful test of whether verbal and perceptual face abilities are associated, we recruited a group of ‘super‐recognizers’ that had been verified as having extremely high levels of perceptual face identification ability based on rigorous prior testing. In Phase 1, we asked super‐recognizers and control participants to describe faces so that people would later be able to recognize them. In Phase 2, we then provided a new set of participants with facial descriptions from Phase 1 and compared their ability to identify faces from descriptions generated by super‐recognizers and control participants. The study was pre‐registered (https://aspredicted.org/VRZ_END).

### Methods

#### Participants

In Phase 1, ‘describer’ participants were either ‘super‐recognizers’ (*N* = 16; *M*_age_ = 39.8 years, 75% female, 75% Caucasian) or ‘controls’ (*N* = 20; *M*_age_ = 54.4 years, 60% female, 100% Caucasian). Super‐recognizers were individuals who in prior testing performed greater than 1.7 standard deviations above the mean on each of three standardized face identification tasks (Cambridge Face Memory Test+ [Russell et al., 2009], Glasgow Face‐Matching Test [Burton et al., 2010], UNSW Face Test [Dunn et al., 2020]). Control participants were individuals who in prior testing performed within one standard deviation of the mean on each of the same standardized face identification tasks (see Table 1). To incentivize participants to generate high‐quality descriptions, we awarded $50, $30, and $20 Amazon vouchers to the participants whose descriptions led to the first, second and third highest identification accuracy in Phase 2 (open to both super‐recognizers and controls). A power analysis indicated our sample size of 36 participants was sufficient to give 80% power (see Supporting Information for details).

**TABLE 1:** Test performance of super‐recognizer and control sample performance on each of the 3 standardized tests (UNSW Face Test, GFMT, and CFMT+) relative to normative test scores (Burton et al., 2010; Dunn et al., 2020; Russell et al., 2009)

	UNSW Face Test, mean (*z* score)	GFMT, mean (*z* score)	CFMT+, mean (*z* score)
Super‐recognizers (*n* = 16)	75.32 (2.78)	100 (1.93)	92.22 (1.978)
Controls (*n* = 20)	58.67 (0.02)	85.15 (0.40)	69.84 (0.02)
Normative test scores	58.57 (−)	81.30 (−)	69.57 (−)

In Phase 2, ‘identifier’ participants were 298 MTurkers from the US and 148 first‐year UNSW Psychology students. MTurkers completed the study in exchange for monetary compensation (US$1.50) while UNSW students completed the study in exchange for course credit. After exclusions for suspected bot performance, study ineligibility, or unusually quick study completion (see Supporting Information for details of exclusions; *N*_MTurkers_ = 24, *N*_UNSW Students_ = 12), the final dataset comprised of 410 identifier participants (MTurkers: *M*_age_ = 35.0 years, 39.9% female, 63% Caucasian; UNSW students: *M*_age_ = 19.6 years, 73.1% female, 52.2% Asian). All included participants in Phase 1 and Phase 2 were native English speakers who had normal or corrected‐to‐normal vision.

#### Materials

##### Face description task (Phase 1)

**FIGURE 5:**
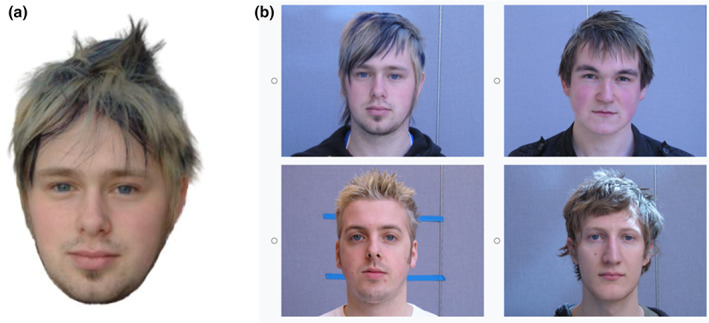
(a) Sample trial from Face Description Task. On each trial, ‘describer’ participants were presented with a face image and asked to ‘Please describe this face. You should describe the face in sufficient detail such that someone else could identify this person solely based on your description’. (b) Sample trial from Identification‐From‐Description Task. On each trial, ‘identifier’ participants were presented with one face description written by a describer in Phase 1 and the line‐up which included the target photo in a random position. Participants were asked ‘Who does the description belong to?’. An example description for this trial is ‘Male, young, pale skin, dyed/bleached dark/light shaggy hair styled to stick up. Blue eyes, medium brown eyebrows. Fairly wide gap between straight eyebrows. Oval face, rounded chin. Plump bottom lip, mouth wide. Tuft of hair under lip, embryonic dark moustache, beard. High forehead’. The individual pictured in the top left is the correct answer

Images in the face description task were taken from the Glasgow Unfamiliar Face Database (GUFD). After excluding faces included in the 40‐item GFMT (Burton et al., 2010), we then selected 10 male, Caucasian faces, aged between 18 and 28 years old. These ‘target’ faces were selected so that the set varied in eye colour, hair colour, and build. During completion of the task, describer participants were shown each of the 10 target faces and asked to ‘describe the face in sufficient detail such that someone else could identify this person solely based on your description’ (see Figure 5a). Our primary dependent variable was description accuracy, indexed by the number of identifiers in Phase 2 who correctly identified the target face with the description over the total number of identifiers in Phase 2 who were given the description.

##### Identification‐from‐description task (Phase 2)

This task was created using target images from Phase 1, and additional images selected from the GUFD to be used as line‐up distractors. The experimenter selected three new faces from the GUFD, who were not included in the 40‐item GFMT (Burton et al., 2010), to serve as distractors for each ‘target’ identity by matching basic demographics (e.g., gender, age, build) and who appeared similar to the target for at least two facial features (e.g., hair colour, eye colour, build, presence of facial hair). The three selected ‘distractor’ images were then placed in an array along with a new image of the target face that had not been used to generate the Phase 1 descriptions, to create target present line‐ups for each target face. The fairness of these line‐ups was pilot tested by showing each to a small group of volunteers who were asked to pick the most distinctive face from the line‐up. Line‐ups were considered fair if, on average, the target identity was not rated as the most distinctive face in the line‐up.

During completion of the task, identifier participants were given 10 descriptions of faces provided by Phase 1 participants. The allocation of descriptions to participants was randomized, however, and was designed such that each description from Phase 1 was allocated to at least 10 different identifiers (post‐data exclusions). For each of the 10 descriptions, identifiers were presented with an array of four faces. On each trial they were instructed to read each description and identify the ‘target’ individual (see Figure 5b). They were additionally prompted with the instruction ‘Note that the descriptions are of each person's face, not necessarily exactly how they appear in these photos’. Although our primary dependent variable was description accuracy, we were additionally interested in the overall accuracy of identifiers, indexed by the percentage of correct identifications in the Identification‐from‐Description Task.

##### Glasgow face‐matching task (GFMT; Phase 2)

This experiment was primarily designed to examine the influence of perceptual face identification ability on verbal description accuracy (i.e., describer ability). However, there was also an opportunity to examine individual differences in identifier ability and so we included the standard 40‐item GFMT (Burton et al., 2010) as a measure of face identification ability in the Australian subset of participants.

#### Procedure

Both Phase 1 and Phase 2 were completed online. In Phase 2, UNSW student identifier participants also completed the GFMT after the Identification‐from‐Description Task. All participants provided information on their highest level of educational attainment and current job occupation as an index for their verbal literacy abilities (Gustafsson, 2016). The average time taken to complete the study was 84.5 min for describers and 15 min for identifiers.

### Results

#### Accuracy

**FIGURE 6:**
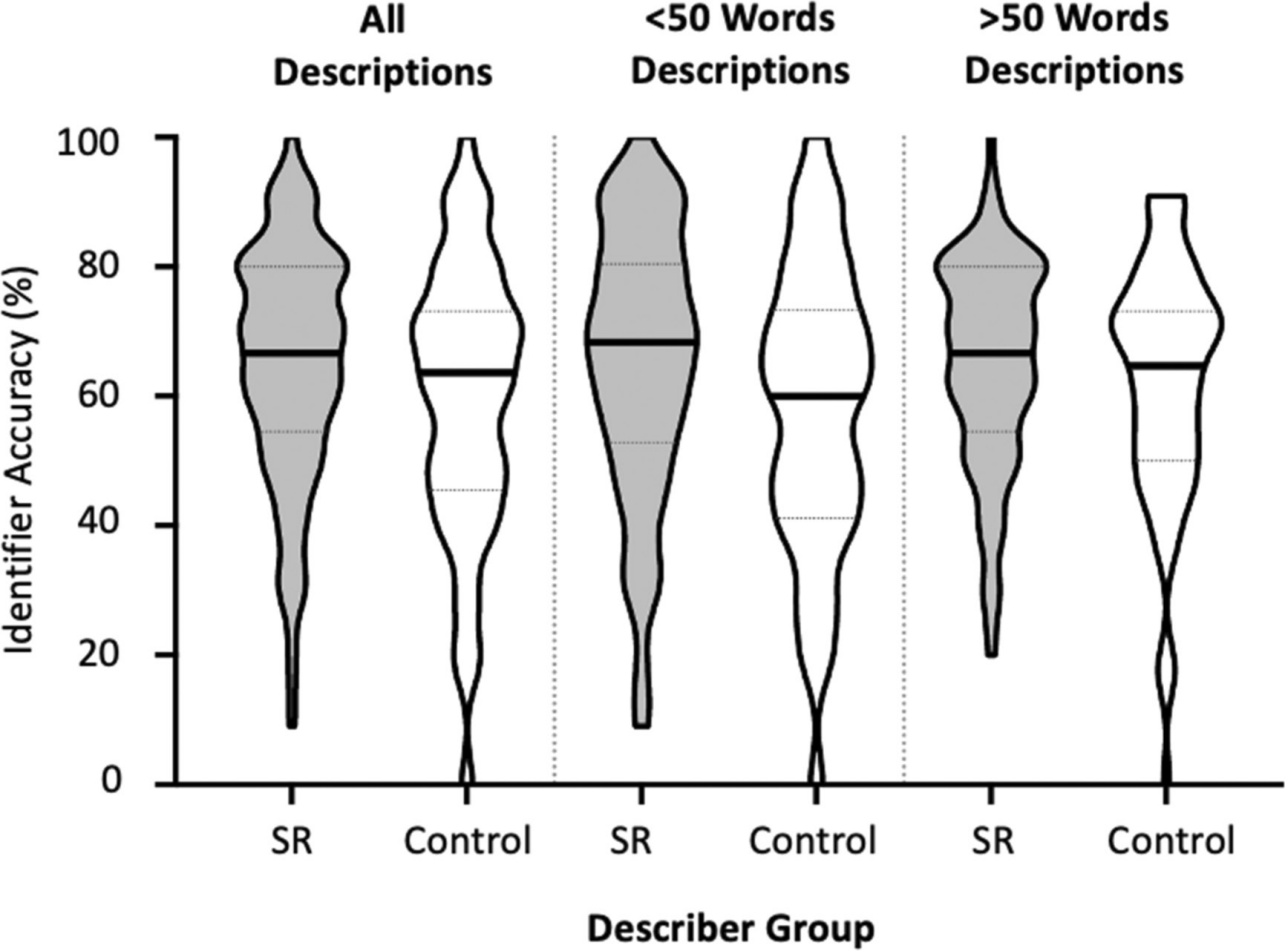
Identifier accuracy is shown on the *Y*‐axis as a function of description type, that is, whether descriptions are generated by super‐recognizer (SR) or control participants. The three different panels show identifier accuracy for all descriptions, short descriptions (<50 words), and long descriptions (>50 words). The solid line on each violin plot represents the median accuracy. The area between the dotted lines on each violent plot represents the interquartile range

Overall accuracy scores of individual participants on the Identification‐from‐Description Task are shown in Figure 6 (left). Across conditions, overall accuracy was 62.0% (*SD* = 22.7) which is substantially greater than chance (25%). Identifiers were on average 5.4% more accurate at identifying faces when using descriptions generated by super‐recognizers (*M* = 65.0%, *SD* = 18.4) than controls (*M* = 59.6%, *SD* = 21.2), and this difference was statistically significant, *t*(358) = 2.52, *p* = .012, Cohen's *d* = .27 [95% CI: 2.495, 8.305].

In post‐hoc analysis, we found that super‐recognizers used approximately 200 more total words on average when describing the 10 target identities (*M* = 658.8, *SD* = 404.7) than controls (*M* = 441.0, *SD* = 194.6), *t*(20.51) = 1.98, *p* = .062, Glass' *delta* = .54 [95% CI: −11.574, 447.174] (NB: we have provided effect size in Glass' *delta* here given the non‐equal variance between groups, but for comparison Cohen's *d* = .69). Given the discrepancy in average total words used per group, we compared super‐recognizer and control descriptions separately for short (<50 words) and long (>50 words) descriptions, based on a median split of description length. For short descriptions, identifiers were on average 7.1% more accurate when given a super‐recognizer's description (*M* = 65.0, *SD* = 21.0) compared to when they were given a control participant's description (*M* = 58.2, *SD* = 22.4), and this difference was statistically significant, *t*(199) = 2.11, *p* = .036, Cohen's *d* = .31 [95% CI: 2.542, 11.058]. In contrast, when considering only longer descriptions (>50 words), there was no difference in identifier accuracy when they were given super‐recognizer descriptions (*M* = 65.0, *SD* = 16.2) and control descriptions (*M* = 62.2, *SD* = 18.9), *t*(155) = 0.989, *p* = .324, Cohen's *d* = .16 [95% CI: −1.109, 6.709]. We also examined whether there were qualitative differences between super‐recognizer and control descriptions in terms of facial features discussed; however, no meaningful differences were observed (see Supporting Information).

As evident from visual inspection of Figure 6, there was large variation in the accuracy with which individual participants identified faces from descriptions. While some of this is likely to stem from the unique set of 10 descriptions provided to each participant, we also tested whether performance was associated with perceptual face identification ability of the identifier participants. There was a weak positive correlation between identifier performance on the Identification‐From‐Description Task and their GFMT scores, *r*_*s*_(132) = .233, *p* = .007.

### Discussion

Study 2 is consistent with Study 1 in showing that people can identify individuals from facial descriptions with reasonable accuracy. Identifiers achieved 62% accuracy on average despite having to use their allocated descriptions for selecting targets from an array of four similar looking faces, which included a different image of the target form that used to generate the description. This was a more challenging task than the pairwise matching in Study 1.

We additionally found that participants could more accurately recognize faces from descriptions written by super‐recognizers than those written by control participants, on average. In contrast to Study 1, this suggests that the ability to describe faces is at least partially related to an individual's perceptual face identification ability. This super‐recognizer description advantage was most evident for concise descriptions, showing that it was driven by differences in the quality of descriptions, rather than length. One possibility is that super‐recognizers are better attuned to what perceptual information is most likely to support identification of an individual and can tailor their facial descriptions accordingly, even when giving concise descriptions. Indeed, previous work has shown that super‐recognizers demonstrate enhanced ability to extract facial information relative to normative controls, such as making more accurate identifications with short image exposures (White et al., 2015) or when faces are disguised (Davis & Tamonytė, 2017). Our work provides preliminary evidence that super‐recognizers can not only extract facial information more effectively but are also more effective at verbally transmitting the most relevant identifying information to someone else.^1^

We also observed large variation in our identifier participants' ability to use facial descriptions for identification, and these were associated with participants' face identity processing ability as measured by the GFMT. However, this analysis was not the main motivation of the study and so we designed a third study to specifically examine the relationship between perceptual face identification ability and people's ability to identify faces from written descriptions.

## STUDY 3

In Study 2, we found preliminary evidence for an association between face identification ability and the ability of ‘identifiers’ to pick faces from a line‐up based on a description. In Study 3, we sought to further investigate this relationship and also examine the relationship between description quality and identifier ability. To test these questions, we recruited a new cohort of super‐recognizers and normative controls who were tasked with identifying faces from facial descriptions, where such descriptions were either ‘good’ or ‘bad’ (as determined by how often the descriptions led to correct identification of the target in Study 2).

### Method

#### Participants

Thirty‐six controls and 36 super‐recognizers completed the study. None had participated in the previous studies. Four control participants and 6 super‐recognizer participants were excluded due to not being Native English speakers. Thus, the final dataset comprised of 32 controls (*M*_age_ = 53.3 years, 84.4% female, 90.6% Caucasian) and 30 super‐recognizers (*M*_age_ = 39.3 years, 70% female, 73.3% Caucasian).

#### Design & procedure

The study was a 2 × 2 between‐subjects design, with factors of Group (super‐recognizer, control) and Description Quality (good, bad). Description quality was manipulated by selecting the three best and three worst descriptions for each target face from Study 2 (i.e., the descriptions which gave rise to the highest or lowest identification rates respectively; see Supporting Information for descriptions). Participants were randomly allocated to a description quality condition (Good: *N*_SR_ = 13, *N*_Control_ = 17; Bad: *N*_SR_ = 17, *N*_Control_ = 15).

Participants completed the Identification‐From‐Description Task (see the Materials section of Study 2 for detailed information about this task). On each trial, participants were randomly allocated one face description that was congruent with their description quality condition (i.e., good or bad). They were additionally prompted with the instruction ‘Note that the descriptions are of each person's face, not necessarily exactly how they appear in these photos’. The order of trials was randomized for each participant.

### Results & discussion

**FIGURE 7:**
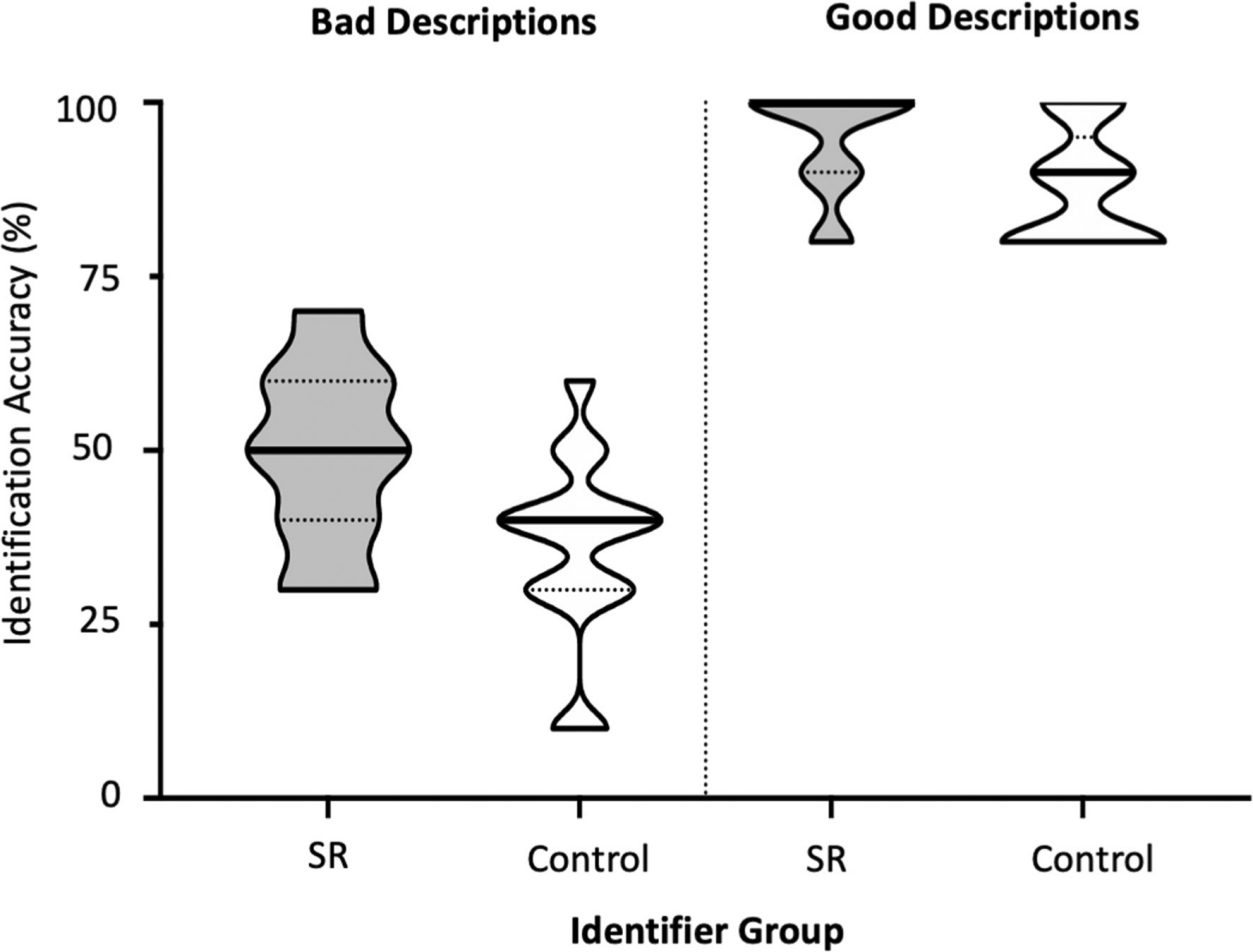
Mean identification accuracy (% correct) on the identification‐from‐description task as a function of identifier group (super‐recognizer [SR] or control). The two different panels show identification accuracy for bad descriptions and good descriptions. The solid line on each violin plot represents the median accuracy. The area between the dotted lines on each violent plot represents the interquartile range

Identification accuracy in Study 3 is shown in Figure 7. We conducted a two‐way ANOVA to examine the relationship between Identifier Group (super‐recognizer or control) and Description Quality (good or bad) on the accuracy of identification decisions. There was a main effect of Description Quality, *F*(1, 60) = 294.02, *p* < .001, *η* ^2^ = .835, with individuals achieving poorer identification accuracy when given bad descriptions (*M* = 42.7%, *SD* = 13.3) compared to when they were given good descriptions (*M* = 91.1%, *SD* = 8.04). The main effect of Identifier Group was also significant, *F*(1, 60) = 13.02, *p* = .001, *η* ^2^ = .183, with super‐recognizers (*M* = 72.0%, *SD* = 10.4) outperforming controls (*M* = 61.8%, *SD* = 10.9). The interaction of identification ability and description type was not significant, *F*(1, 60) = 1.30, *p* = .259, *η* ^2^ = .022.

The results show that the ability to use facial descriptions for identification is driven in large part by the quality of the description provided. However, they also indicate that super‐recognizers are better able to make identifications based on facial descriptions than individuals with normative levels of face identification ability. Further to Study 2, this suggests super‐recognizers may be better able to extract diagnostic perceptual information from facial descriptions to make identifications, in addition to being better at selecting and describing the identifying features of a face.^2^

## GENERAL DISCUSSION

We conducted three experiments to better understand how people communicate about faces and the utility of facial descriptions for identification purposes. Across these studies, we consistently showed that individuals can communicate about faces with reasonable accuracy, regardless of whether such communication occurred bidirectionally in a live matching context (Study 1) or unidirectionally when isolating the abilities of making facial descriptions from using facial descriptions for identification (Study 2 and Study 3). We also consistently showed evidence of large variation in face communication efficacy, which were associated with the face identification abilities of the ‘describer’ (Study 2) and the ‘identifier’ (Study 3). This association was subtle, likely reflecting that face communication relies on other abilities, for example verbal skill.

Prior work in this area shows above‐chance identification performance based on verbal descriptions (Fallshore & Schooler, 1995; Kramer & Gous, 2020). Study 1 improved understanding of face communication by showing that difficulties in describing faces for identification purposes are not ameliorated by allowing naturalistic communication. This study also showed that the decrease in performance in face‐matching when using verbal descriptions, compared to perceptual matching, was entirely driven by an increase in false positive errors. Although the reason for this bias is not clear, it is nonetheless practically important, suggesting for example that when searching for persons of interest in criminal investigation, descriptions could elicit many spurious leads.

To our knowledge, this is the first set of studies to explore how individual differences in face perception relate to face communication. Prior work has focused on the accuracy of face communication (Fallshore & Schooler, 1995; Kramer & Gous, 2020). Here, we found that face identification skill contributed to variance in face communication performance. When examining the face processing abilities of super‐recognizers, we found a sizeable performance advantage in both describing faces and using face descriptions for the purpose of identification, as compared to individuals with normative levels of face identification skill. This result therefore broadens the scope of super‐recognizers' abilities. While some studies have found evidence of supra‐modal person identification abilities that extend to voice recognition (Jenkins et al., 2021), general object processing (Bobak et al., 2016) and fingerprint matching (Towler et al., 2021), our result is the first to show that super‐recognizers demonstrate enhanced ability beyond purely perceptual tasks. Future research may like to explore how the full spectrum of individual differences in face perception relate to face communication.

We also aimed to better understand what aspects of facial descriptions were most effective for identification. In Study 1, we found no association between the facial features discussed by participants, nor overall comments about facial appearance, and face communication accuracy. Similarly, in Study 2, we found no obvious differences in the content of super‐recognizer and control descriptions despite differences in the mean accuracy of their descriptions. This extends work by Ritchie et al. (2022) who found no relationship between facial features discussed and the accuracy of dyads on a face‐matching task when both images were in view simultaneously. In the context of face communication specifically, Kramer and Gous (2020) found that discussion of the eyes, nose, mouth, hair, ears and face shape were not associated with identification accuracy. That accuracy was similarly not associated with the discussion of other facial features in our study (e.g., facial hair, jawline, cheeks, facial marks) shows that the inclusion/exclusion of particular facial features in face descriptions is not what makes them effective for identification purposes. This raises the question of what makes for an effective face description.

One possible explanation is that the content of facial descriptions is less important than the communication context they are exchanged in, including the interpersonal dynamics unique to each dyad in Study 1 (e.g., rapport, familiarity, conversational approach, propensity for information seeking vs. information giving). Indeed, in many other areas of communication, interpersonal factors are predictive of performance, including for negotiation outcomes, goal achievement, and social outcomes including likeability and attractiveness (Curhan & Pentland, 2007; Leung & Bond, 2001; Martin & Dowson, 2009). In the context of face communication, one potential interpersonal contributor to accuracy is the degree of familiarity between describers and identifiers. In our experiments, participants were unfamiliar (i.e., had no personal relationship) with their study partner (Study 1) or with the person they were generating face descriptions for or receiving descriptions from (Study 2 and Study 3). However, familiarity with the other person involved in face communication may confer benefits to task performance, including a nuanced understanding of each other's vocabulary, particularly for use of ambiguous terms. In support of this idea, evidence suggests that linguistic similarities predict friendship forming, and that friends, ‘new’ couples, and married couples exhibit linguistic convergence over time (Anolli & Balconi, 2005; Brinberg & Ram, 2021; Kovacs & Kleinbaum, 2020). Consequently, future research could examine whether the nature of the relationship between interlocuters in face communication affects the usefulness of descriptions for identification.

Another possibility is that qualitative aspects of face descriptions are more important to face communication accuracy than quantitative properties (e.g., description length, number of features discussed). For example, in creative writing, vivid descriptions tend to evoke richer mental imagery of described stimuli than list‐based descriptors or those that are more precise (Jajdelska et al., 2010). It has also been argued that fictional character descriptions which facilitate the reader's connection with the sensory experience of the protagonist (e.g., based on metaphor) are more likely to elicit an emotional reaction and compel an individual to continue reading, as compared with narrative summary (Ingermanson & Economy, 2009). However, whether vivid or emotionally salient descriptions are more accurate for identification purposes is unknown. Consequently, future research could further explore the qualitative aspects distinguishing good and poor face descriptions, which in turn may inform avenues for training in face communication.

The goal of the present work was to explore the accuracy of face communication and examine its relationship to individual differences in face identification. Given the exploratory nature of our work, we did not seek to develop a psychometric test optimized for the measurement of individual differences in face communication. Nonetheless, the reliability of measures is an important consideration in the interpretation of results. In post‐hoc analysis, we found relatively good internal reliability of identifiers (*α* = .621 and *α* = .815 for identifiers in Study 2 and Study 3 respectively) while for real‐time face communicators and describers internal reliability was below accepted psychometric thresholds (Study 1: *α* = .316 for the Verbal GFMT; Study 2: *α* = .134 for describers). Consequently, it is unclear if variation in performance on face description measures reflects individual differences or test unreliability.

The fact we see higher internal reliability for identifiers than describers suggests that the way people use descriptions for identification may be more consistent than the way people generate descriptions. It might also suggest that researchers' ability to measure an individual's face description ability, using the novel approach we describe here, is confounded by idiosyncratic properties of the person using that description for identification – and/or the faces being described – rather than intrinsic properties of the description or describer. Future research aiming to develop psychometric tests for face communication could therefore aim to redress this difficulty by using more diverse stimuli, and also perhaps by constraining the descriptions more than the free description method used here (e.g., with a rating scale procedure).

Another important consideration in the interpretation of our results is that in the present set of studies all images used were highly controlled in that they were studio quality, front‐facing photos taken minutes apart. Although investigating performance under controlled image conditions is a useful first step in understanding face communication, such conditions may not reflect the real‐world nature of face communication. For example, when identifying someone from a description in the real world, the described individual may differ substantially from the image which the description is based on due to factors such as ageing, disguise, or routine appearance modification (e.g., getting a haircut, applying make‐up). In purely perceptual tasks, identification accuracy falls when task difficulty is increased by increasing image variation (Noyes & Jenkins, 2019; White et al., 2015). Consequently, future studies could examine the decrement in face communication caused by more naturalistic variation in images.

In sum, our findings show that individuals can communicate about faces and there is large variation in this ability. Additionally, perceptual face identification skill partly explains why some facial descriptions are better than others as well as why certain individuals are more accurate at using facial descriptions for identification. While many prior studies in face identification have investigated the effect of perceptual task demands on performance (Hancock et al., 2000), we have shown here that verbal processes are another important contributor to accuracy, which has implications for a number of applied tasks. We recommend that legal and forensic stakeholders temper decision‐making based on evidence that requires verbal communication of perceptual face information as deriving face identity from face descriptions generates an especially high rate of false positives relative to standard identity processing. Moving forward, to obtain a full understanding of the complexities in face identification, it will be important for research to consider not only the impact of perceptual demands but also verbal processes on performance.

## AUTHOR CONTRIBUTIONS

**Rebecca Louise Tyler:** Conceptualization; data curation; formal analysis; methodology; visualization; writing – original draft; writing – review and editing. **Alice Towler:** Conceptualization; methodology; supervision; writing – review and editing. **Richard Kemp:** Conceptualization; methodology; supervision; writing – review and editing. **David White:** Conceptualization; methodology; supervision; writing – review and editing.

## CONFLICT OF INTEREST

The authors declare no potential conflict of interest.

## Supporting information


Appendix S1.

Data S1.

## Data Availability

The data that support the findings of this study and Supporting Information (additional analyses and study materials referred to in‐text) are openly available at https://osf.io/wxav2/?view_only=d6d1ca2f507e4db58575b6f4ea135db0.

## Footnotes

^1^
Given Study 1 found that the Verbal GFMT had low internal reliability, we performed similar post‐hoc reliability analyses of face communication measures used in Study 2. The internal reliability for describers *α* = .134 and for identifiers was *α* = .621. We return to this finding in the General Discussion.

^2^
Similar to Study 1 and Study 2, we completed post‐hoc internal reliability analyses. Internal reliability for identifiers was *α* = .815. This appears to be driven by a marginally higher internal reliability statistic for controls (*α* = .832) than for super‐recognisers (*α* = .794). We return to this finding in the General Discussion.

## References

[bjop12610-bib-0001] Altman, D. G. (1990). Practical statistics for medical research. CRC Press.

[bjop12610-bib-0002] Anolli, L. , & Balconi, M. (2005). Topic variability and linguistic convergence in marital couples' speech as related to specific attachment style. Psychological Reports, 96(1), 83–106. 10.2466/pr0.96.1.83-106 15825910

[bjop12610-bib-0003] Balsdon, T. , Summersby, S. , Kemp, R. I. , & White, D. (2018). Improving face identification with specialist teams. Cognitive Research: Principles and Implications, 3(25), 1–13. 10.1186/s41235-018-0114-7 29984300 PMC6021458

[bjop12610-bib-0004] Block, K. K. , & Beckett, K. D. (1990). Verbal descriptions of skill by specialists and nonspecialists. Journal of Teaching in Physical Education, 10(1), 21–37. 10.1123/jtpe.10.1.21

[bjop12610-bib-0005] Bobak, A. K. , Bennetts, R. J. , Parris, B. A. , Jansari, A. , & Bate, S. (2016). An in‐depth cognitive examination of individuals with superior face recognition skills. Cortex, 82, 48–62. 10.1016/j.cortex.2016.05.003 27344238

[bjop12610-bib-0006] Brinberg, M. , & Ram, N. (2021). Do new romantic couples use more similar language over time? Evidence from intensive longitudinal text messages. Journal of Communication, 71(3), 454–477. 10.1093/joc/jqab012 34335083 PMC8315721

[bjop12610-bib-0007] Bruce, V. , Henderson, Z. , Newman, C. , & Burton, A. M. (2001). Matching identities of familiar and unfamiliar faces caught on CCTV images. Journal of Experimental Psychology: Applied, 7(3), 207–218. 10.1037/1076-898X.7.3.207 11676099

[bjop12610-bib-0008] Burton, A. M. , & Jenkins, R. (2011). Unfamiliar face perception. In The Oxford handbook of face perception (pp. 287–306). Oxford University Press.

[bjop12610-bib-0009] Burton, A. M. , White, D. , & McNeill, A. (2010). The Glasgow face matching test. Behavior Research Methods, 42(1), 286–291. 10.3758/BRM.42.1.286 20160307

[bjop12610-bib-0010] Chaturvedi, S. , & Shweta, R. (2015). Evaluation of inter‐rater agreement and inter‐rater reliability for observational data: an overview of concepts and methods. Journal of the Indian Academy of Applied Psychology, 41(3), 20–27.

[bjop12610-bib-0011] Connelly, M. (2012). The sundance writer: A rhetoric, reader, and research guide, brief. Cengage Learning.

[bjop12610-bib-0012] Curhan, J. R. , & Pentland, A. (2007). Thin slices of negotiation: Predicting outcomes from conversational dynamics within the first 5 minutes. Journal of Applied Psychology, 92(3), 802–811. 10.1037/0021-9010.92.3.802 17484559

[bjop12610-bib-0013] Davis, J. P. , & Tamonytė, D. (2017). Masters of disguise: super‐recognisers' superior memory for concealed unfamiliar faces. 2017 Seventh International Conference on Emerging Security Technologies (EST).

[bjop12610-bib-0014] DeGutis, J. M. , Chiu, C. , Grosso, M. E. , & Cohan, S. (2014). Face processing improvements in prosopagnosia: Successes and failures over the last 50 years. Frontiers in Human Neuroscience, 8(561), 1–14. 10.3389/fnhum.2014.00561 25140137 PMC4122168

[bjop12610-bib-0015] Dowsett, A. J. , & Burton, A. M. (2015). Unfamiliar face matching: Pairs out‐perform individuals and provide a route to training. British Journal of Psychology, 106(3), 433–445. 10.1111/bjop.12103 25393594

[bjop12610-bib-0016] Duchaine, B. , & Nakayama, K. (2006). The Cambridge Face Memory Test: Results for neurologically intact individuals and an investigation of its validity using inverted face stimuli and prosopagnosic participants. Neuropsychologia, 44(4), 576–585. 10.1016/j.neuropsychologia.2005.07.001 16169565

[bjop12610-bib-0017] Dunn, J. D. , de Lima Varela, V. P. , Nicholls, V. I. , Papinutto, M. , White, D. , & Miellet, S. (2022). Face information sampling in super‐recognizers. Psychological Science, 33(9), 1615–1630. 36044042 10.1177/09567976221096320

[bjop12610-bib-0018] Dunn, J. D. , Summersby, S. , Towler, A. , Davis, J. P. , & White, D. (2020). UNSW Face Test: A screening tool for super‐recognizers. PLoS One, 15(11), e0241747. 10.1371/journal.pone.0241747 33196639 PMC7668578

[bjop12610-bib-0019] Fallshore, M. , & Schooler, J. W. (1995). Verbal vulnerability of perceptual expertise. Journal of Experimental Psychology: Learning, Memory, and Cognition, 21(6), 1608–1623. 10.1037/0278-7393.21.6.1608 7490581

[bjop12610-bib-0020] Feldt, L. S. (1961). The use of extreme groups to test for the presence of a relationship. Psychometrika, 26(3), 307–316.

[bjop12610-bib-0021] Gustafsson, J.‐E. (2016). Lasting effects of quality of schooling: Evidence from PISA and PIAAC. Intelligence, 57, 66–72. 10.1016/j.intell.2016.05.004

[bjop12610-bib-0022] Hancock, P. J. , Bruce, V. , & Burton, A. M. (2000). Recognition of unfamiliar faces. Trends in Cognitive Sciences, 4(9), 330–337. 10.1016/S1364-6613(00)01519-9 10962614

[bjop12610-bib-0023] Hughson, A. L. , & Boakes, R. A. (2009). Short Article: Passive perceptual learning in relation to wine: Short‐term recognition and verbal description. Quarterly Journal of Experimental Psychology, 62(1), 1–8. 10.1080/17470210802214890 18622887

[bjop12610-bib-0024] Ingermanson, R. , & Economy, P. (2009). Writing fiction for dummies. John Wiley & Sons.

[bjop12610-bib-0025] Jajdelska, E. , Butler, C. , Kelly, S. , McNeill, A. , & Overy, K. (2010). Crying, moving, and keeping it whole: What makes literary description vivid? Poetics Today, 31(3), 433–463. 10.1215/03335372-2010-002

[bjop12610-bib-0026] Jenkins, R. E. , Tsermentseli, S. , Monks, C. P. , Robertson, D. J. , Stevenage, S. V. , Symons, A. E. , & Davis, J. P. (2021). Are super‐face‐recognisers also super‐voice‐recognisers? Evidence from cross‐modal identification tasks. Applied Cognitive Psychology, 35(3), 590–605. 10.1002/acp.3813

[bjop12610-bib-0027] Kovacs, B. , & Kleinbaum, A. M. (2020). Language‐style similarity and social networks. Psychological Science, 31(2), 202–213. 10.1177/0956797619894557 31877069

[bjop12610-bib-0028] Kramer, R. S. , & Gous, G. (2020). Eyewitness descriptions without memory: The (f) utility of describing faces. Applied Cognitive Psychology, 34(3), 605–615. 10.1002/acp.3645

[bjop12610-bib-0029] Leung, S. K. , & Bond, M. H. (2001). Interpersonal communication and personality: Self and other perspectives. Asian Journal of Social Psychology, 4(1), 69–86. 10.1111/1467-839X.00076

[bjop12610-bib-0030] Lindsay, R. , Mansour, J. K. , Bertrand, M. I. , Kalmet, N. , & Melsom, E. I. (2011). Face recognition in eyewitness memory. In The Oxford handbook of face perception (pp. 307–328). Oxford University Press.

[bjop12610-bib-0031] Mäntylä, K. (2004). Idioms and language users: The effect of the characteristics of idioms on their recognition and interpretation by native and non‐native speakers of English. Jyväskylän yliopisto.

[bjop12610-bib-0032] Marina, V. , & Snuviškiene, G. (2005). Error analysis of scientific papers written by non‐native speakers of English. Transport, 20(6), 274–279. 10.1080/16484142.2005.9638031

[bjop12610-bib-0033] Martin, A. J. , & Dowson, M. (2009). Interpersonal relationships, motivation, engagement, and achievement: Yields for theory, current issues, and educational practice. Review of Educational Research, 79(1), 327–365. 10.3102/0034654308325583

[bjop12610-bib-0035] Meissner, C. A. , Sporer, S. L. , & Schooler, J. W. (2007). Person descriptions as eyewitness evidence. In R. C. L. Lindsay , D. F. Ross , J. Don Read , & M. P. Toglia (Eds.), Handbook of eyewitness psychology: Memory for people (Vol. 2, pp. 1–34). Psychology Press.

[bjop12610-bib-0036] Nickerson, R. S. (1998). Confirmation bias: A ubiquitous phenomenon in many guises. Review of General Psychology, 2(2), 175–220. 10.1037/1089-2680.2.2.175

[bjop12610-bib-0037] Noyes, E. , & Jenkins, R. (2019). Deliberate disguise in face identification. Journal of Experimental Psychology: Applied, 25(2), 280–290. 10.1037/xap0000213 30730157

[bjop12610-bib-0038] Noyes, E. , Phillips, P. J. , & O'Toole, A. J. (2017). What is a super‐recogniser? In Face processing: Systems, disorders and cultural differences (pp. 173–201). Nova Science Publishers Inc.

[bjop12610-bib-0039] Oosterhof, N. N. , & Todorov, A. (2008). The functional basis of face evaluation. Proceedings of the National Academy of Sciences, 105(32), 11087–11092. 10.1073/pnas.0805664105 PMC2516255 18685089

[bjop12610-bib-0040] Ritchie, K. L. , Flack, T. R. , Fuller, E. A. , Cartledge, C. , & Kramer, R. S. S. (2022). The pairs training effect in unfamiliar face matching . Unpublished manuscript. School of Psychology, University of Lincoln. 10.1177/03010066221096987 PMC9203675 35581726

[bjop12610-bib-0041] Russell, R. , Duchaine, B. , & Nakayama, K. (2009). Super‐recognizers: People with extraordinary face recognition ability. Psychonomic Bulletin & Review, 16(2), 252–257. 10.3758/PBR.16.2.252 19293090 PMC3904192

[bjop12610-bib-0042] Ryan, R. S. , & Schooler, J. W. (1998). Whom do words hurt? Individual differences in susceptibility to verbal overshadowing. Applied Cognitive Psychology, 12(7), S105–S125. 10.1002/(SICI)1099-0720(199812)12:7<S105::AID-ACP597>3.0.CO;2-V

[bjop12610-bib-0045] Solomon, G. E. A. (1990). Psychology of novice and expert wine talk. The American Journal of Psychology, 103, 495–517.

[bjop12610-bib-0046] Spence, C. , & Wang, Q. J. (2019). Wine expertise: Perceptual learning in the chemical senses. Current Opinion in Food Science, 27, 49–56. 10.1016/j.cofs.2019.05.003

[bjop12610-bib-0047] Sporer, S. L. (1992). Post‐dicting eyewitness accuracy: Confidence, decision‐times and person descriptions of choosers and non‐choosers. European Journal of Social Psychology, 22(2), 157–180. 10.1002/ejsp.2420220205

[bjop12610-bib-0049] Towler, A. , Dunn, J. D. , Martínez, S. C. , Moreton, R. , Eklöf, F. , Ruifrok, A. , Kemp, R. , & White, D. (2021). Diverse routes to expertise in facial recognition. *psyArXiv* . 10.31234/osf.io/fmznh PMC10349110 37452069

[bjop12610-bib-0050] Towler, A. , Kemp, R. I. , Burton, A. M. , Dunn, J. D. , Wayne, T. , Moreton, R. , & White, D. (2019). Do professional facial image comparison training courses work? PLoS One, 14(2), e0211037. 10.1371/journal.pone.0211037 30759105 PMC6373902

[bjop12610-bib-0051] Towler, A. , Kemp, R. I. , & White, D. (2020). Can face identification ability be trained?: Evidence for two routes to expertise. In Forensic face matching (pp. 89–114). Oxford University Press.

[bjop12610-bib-0052] Towler, A. , White, D. , & Kemp, R. I. (2014). Evaluating training methods for facial image comparison: The face shape strategy does not work. Perception, 43(2–3), 214–218. 10.1068/p7676 24919354

[bjop12610-bib-0053] Van Koppen, P. J. , & Lochun, S. K. (1997). Portraying perpetrators; the validity of offender descriptions by witnesses. Law and Human Behavior, 21(6), 661–685. 10.1023/A:1024812831576

[bjop12610-bib-0054] Van Paridon, J. , Ostarek, M. , Arunkumar, M. , & Huettig, F. (2021). Does neuronal recycling result in destructive competition? The influence of learning to read on the recognition of faces. Psychological Science, 32(3), 459–465. 10.1177/0956797620971652 33631074

[bjop12610-bib-0055] White, D. , & Burton, A. M. (2022). Individual differences and the multidimensional nature of face perception. Nature Reviews Psychology, 1(5), 287–300. 10.1038/s44159-022-00041-3

[bjop12610-bib-0056] White, D. , Phillips, P. J. , Hahn, C. A. , Hill, M. , & O'Toole, A. J. (2015). Perceptual expertise in forensic facial image comparison. Proceedings of the Royal Society B, 282(1814), 1–8. 10.1098/rspb.2015.1292 PMC4571699 26336174

[bjop12610-bib-0057] Wilmer, J. B. , Germine, L. , Chabris, C. F. , Chatterjee, G. , Williams, M. , Loken, E. , Nakayama, K. , & Duchaine, B. (2010). Human face recognition ability is specific and highly heritable. Proceedings of the National Academy of Sciences, 107(11), 5238–5241. 10.1073/pnas.0913053107 PMC2841913 20176944

