# Supplementary material for: Let's talk about faces: Identifying faces from verbal descriptions
Source: Br J Psychol. 2022 Nov 4;114(1):262–81. doi: 10.1111/bjop.12610 (PMC10100156; doi:10.1111/bjop.12610)
Supplement: Supplementary file 1 — Appendix S1. [file BJOP-114-262-s001.docx]

**Let’s Talk About Faces: Identifying Faces from Verbal Descriptions
Supplementary Materials**

**Study 1**

**Correlations between Verbal GFMT and best/worst performing individual on Perceptual GFMT:**

Best-performing: r_s_ = .184, p = .196

Worst-performing: r_s_ = .065, p = .650

**Correlations between factors and Verbal GFMT scores:**

*Table 1*. Spearman’s rho (*r_s_*) correlation coefficients between possible predictors of Verbal GFMT performance and Verbal GFMT accuracy (before Bonferroni correction)

| **Predictor** | ***r_s_* value** | **p-value** |
| --- | --- | --- |
| Pairs’ Average Perceptual GFMT Performance | .115 | .420 |
| Best Performing Individual Perceptual GFMT Performance | .184 | .196 |
| Worst Performing Individual Perceptual GFMT Performance | .065 | .650 |
| Total Time Taken on Verbal GFMT | .028 | .849 |
| Mean Time Taken on Match Trials of Verbal GFMT | .124 | .393 |
| Mean Time Taken on Non-Match Trials of Verbal GFMT | -.019 | .897 |
| Total Facial Feature Mentions | .097 | .504 |
| Total Internal Facial Feature Mentions | -.048 | .738 |
| Total External Facial Feature Mentions | .028 | .847 |
| Total Holistic Judgements | .033 | .822 |

*Table 2*. Spearman’s rho (*r_s_*) correlation coefficients between individual feature mentions and verbal GFMT accuracy (before Bonferroni correction).

|  | Verbal GFMT  All Trials | | Verbal GFMT Match Trials | | Verbal GFMT Mismatch Trials | |
| --- | --- | --- | --- | --- | --- | --- |
| Facial Feature | r_s_ value | p-value | r_s_ value | p-value | r_s_ value | p-value |
| Eyes | -.011 | .942 | -.019 | .898 | .059 | .686 |
| Eyebrows | .094 | .518 | -.044 | .760 | -.085 | .557 |
| Nose | -.0.27 | .855 | -.016 | .912 | .031 | .829 |
| Mouth | -.058 | .691 | -.032 | .827 | -.050 | .732 |
| Forehead | .150 | .299 | .061 | .673 | -.070 | .627 |
| Chin | 0.25 | .866 | -.128 | .375 | .004 | .977 |
| Jawline | -.020 | .892 | -.237 | .098 | -.149 | .303 |
| Cheeks | .135 | .351 | .053 | .714 | .059 | .686 |
| Faceshape | -.230 | .108 | -.033 | .821 | -.219 | .127 |
| Facial Hair | -.078 | .589 | -.150 | .297 | -.076 | .598 |
| Hair | .044 | .762 | .005 | .975 | -.239 | .095 |
| Ears | .139 | .337 | -.308* | .030 | -.069 | .632 |
| Facial Marks | -.367** | .009 | -.009 | .952 | .044 | .759 |

*=significant at the .05 level (2-tailed). **=significant at the .01 level (2-tailed).

**Study 2**

**Power analysis**

Power analysis completed in <https://clincalc.com/stats/samplesize.aspx>

2 independent groups, continuous DV.

Group 1 *M* = 80, SD = 10

Group 2 *M* = 70

Group means pertain to anticipated performance accuracy on the Identification-from-Description Task for identifiers given facial descriptions written by super-recognisers (Group 1) versus identifiers given facial descriptions written by controls (Group 2). In prior research on face expertise in super-recognition, it is well-known that super-recognisers outperform normative controls. In fact, the cut-off for classification as a super-recogniser is typically performance approximately 1.5-2 standard deviations above the mean on 1 or more standardised test of face recognition ability (e.g., Russell et al., 2009; Ramon, 2021; Bobak et al., 2016). Here, we chose a 1 standard deviation accuracy advantage as a conservative estimate of the size of a super-recogniser effect we may see in a task requiring abilities outside core face processing systems (such as language).

Alpha = 0.05

Power = 80%

Required sample size = 16 per group.

**Details of Identifier Participant Exclusions**

5 x MTurkers excluded for being non-Native English speakers

12 x SONA-1 participants excluded for being non-Native English speakers

15 x MTurkers were excluded for completing the study in <5 minutes

1 x SONA-1 participant was excluded for completing the study in <5 minutes

3 x MTurkers excluded for suspected bot performance (1 answered ‘1810’ for occupation, 1 answered ‘515’ for age, 1 answered ’49,999’ for occupation)

**SR and Control Word/Feature Frequency**

| **200 Most Used Words** | **% of SR descriptions (Combined total = 73.88%)** | **% of Control Descriptions (Combined total = 79.28%)** | **SR Rank of Word** | **Control Rank of Word** |
| --- | --- | --- | --- | --- |
| hair | 2.77 | 3.636602 | 1 | 1 |
| eyes | 2.13 | 2.835762 | 3 | 2 |
| nose | 2.16 | 2.612577 | 2 | 3 |
| face | 1.93 | 2.336878 | 4 | 4 |
| brown | 1.67 | 2.061179 | 6 | 5 |
| chin | 1.74 | 1.378495 | 5 | 10 |
| eyebrows | 1.27 | 1.667323 | 10 | 7 |
| lip | 1.52 | 1.352238 | 7 | 12 |
| short | 1.17 | 1.680452 | 12 | 6 |
| top | 1.41 | 1.404753 | 9 | 9 |
| blue | 1.14 | 1.627938 | 13 | 8 |
| left | 1.24 | 1.378495 | 11 | 11 |
| slightly | 1.48 | 1.089668 | 8 | 18 |
| lips | 1.14 | 1.299724 | 13 | 14 |
| ears | 1.03 | 1.325981 | 17 | 13 |
| small | 1.10 | 0.958383 | 15 | 22 |
| dark | 0.79 | 1.286596 | 21 | 15 |
| thin | 0.75 | 1.273467 | 24 | 16 |
| light | 1.05 | 0.918997 | 16 | 23 |
| right | 0.77 | 1.220953 | 23 | 17 |
| straight | 0.83 | 0.997768 | 19 | 20 |
| stubble | 0.78 | 0.918997 | 22 | 24 |
| bottom | 0.75 | 0.840226 | 24 | 26 |
| side | 0.54 | 1.024025 | 40 | 19 |
| long | 0.68 | 0.840226 | 27 | 27 |
| skin | 0.65 | 0.853354 | 29 | 25 |
| slight | 0.83 | 0.577655 | 19 | 37 |
| medium | 0.73 | 0.643298 | 26 | 34 |
| pale | 0.40 | 0.958383 | 55 | 21 |
| male | 0.56 | 0.748326 | 37 | 31 |
| prominent | 0.54 | 0.761455 | 40 | 29 |
| round | 0.58 | 0.682683 | 34 | 32 |
| very | 0.68 | 0.564527 | 27 | 39 |
| full | 0.46 | 0.80084 | 49 | 28 |
| ear | 0.50 | 0.748326 | 45 | 30 |
| green | 0.64 | 0.577655 | 30 | 36 |
| forehead | 0.60 | 0.617041 | 32 | 35 |
| head | 0.56 | 0.643298 | 37 | 33 |
| facial | 0.59 | 0.53827 | 33 | 42 |
| shaped | 0.54 | 0.459498 | 40 | 49 |
| oval | 0.47 | 0.53827 | 48 | 43 |
| caucasian | 0.34 | 0.564527 | 67 | 38 |
| square | 0.34 | 0.551398 | 67 | 41 |
| around | 0.57 | 0.288828 | 35 | 72 |
| jaw | 0.36 | 0.525141 | 65 | 44 |
| thick | 0.50 | 0.35447 | 45 | 63 |
| length | 0.34 | 0.525141 | 67 | 45 |
| eye | 0.38 | 0.472627 | 61 | 48 |
| upper | 0.52 | 0.301956 | 44 | 71 |
| mouth | 0.46 | 0.367599 | 49 | 61 |
| fringe | 0.28 | 0.551398 | 83 | 40 |
| wide | 0.33 | 0.485756 | 72 | 47 |
| nostrils | 0.37 | 0.433241 | 63 | 52 |
| rounded | 0.39 | 0.380727 | 58 | 58 |
| grey | 0.33 | 0.44637 | 72 | 50 |
| narrow | 0.32 | 0.44637 | 77 | 51 |
| no | 0.34 | 0.406984 | 67 | 55 |
| lower | 0.41 | 0.301956 | 52 | 69 |
| bridge | 0.50 | 0.196928 | 45 | 105 |
| more | 0.40 | 0.288828 | 55 | 73 |
| down | 0.33 | 0.35447 | 72 | 62 |
| white | 0.39 | 0.275699 | 58 | 76 |
| fair | 0.40 | 0.249442 | 55 | 84 |
| from | 0.36 | 0.301956 | 65 | 68 |
| mid | 0.30 | 0.367599 | 80 | 59 |
| large | 0.18 | 0.485756 | 125 | 46 |
| under | 0.33 | 0.301956 | 72 | 70 |
| close | 0.26 | 0.380727 | 90 | 57 |
| jawline | 0.41 | 0.196928 | 52 | 106 |
| complexion | 0.38 | 0.236314 | 61 | 86 |
| shape | 0.39 | 0.183799 | 58 | 115 |
| out | 0.32 | 0.262571 | 77 | 79 |
| blonde | 0.28 | 0.301956 | 83 | 67 |
| red | 0.20 | 0.393856 | 115 | 56 |
| looks | 0.41 | 0.131285 | 52 | 150 |
| between | 0.30 | 0.249442 | 80 | 82 |
| centre | 0.23 | 0.328213 | 103 | 64 |
| colour | 0.23 | 0.328213 | 103 | 65 |
| moustache | 0.19 | 0.367599 | 117 | 60 |
| hairline | 0.33 | 0.183799 | 72 | 112 |
| set | 0.26 | 0.262571 | 90 | 80 |
| sideburns | 0.26 | 0.262571 | 90 | 81 |
| longer | 0.27 | 0.236314 | 88 | 90 |
| like | 0.37 | 0.105028 | 63 | 180 |
| double | 0.34 | 0.131285 | 67 | 145 |
| or | 0.28 | 0.196928 | 83 | 109 |
| quite | 0.25 | 0.223185 | 94 | 98 |
| cheek | 0.24 | 0.236314 | 99 | 85 |
| mole | 0.24 | 0.223185 | 99 | 97 |
| tip | 0.32 | 0.118157 | 77 | 165 |
| some | 0.28 | 0.157542 | 83 | 131 |
| sides | 0.21 | 0.236314 | 111 | 92 |
| growth | 0.06 | 0.406984 | 263 | 54 |
| cheeks | 0.30 | 0.131285 | 80 | 143 |
| towards | 0.25 | 0.183799 | 94 | 116 |
| above | 0.23 | 0.210056 | 103 | 100 |
| almost | 0.23 | 0.210056 | 103 | 101 |
| beard | 0.21 | 0.223185 | 111 | 93 |
| line | 0.21 | 0.223185 | 111 | 96 |
| ginger | 0.25 | 0.170671 | 94 | 118 |
| not | 0.25 | 0.170671 | 94 | 119 |
| middle | 0.23 | 0.183799 | 103 | 114 |
| black | 0.14 | 0.275699 | 143 | 74 |
| visible | 0.26 | 0.118157 | 90 | 167 |
| pink | 0.27 | 0.0919 | 88 | 209 |
| freckles | 0.15 | 0.223185 | 137 | 95 |
| flat | 0.12 | 0.262571 | 166 | 78 |
| wider | 0.28 | 0.065643 | 83 | 264 |
| look | 0.25 | 0.105028 | 94 | 182 |
| cut | 0.13 | 0.236314 | 153 | 87 |
| broad | 0.09 | 0.275699 | 198 | 75 |
| features | 0.23 | 0.118157 | 103 | 155 |
| one | 0.23 | 0.118157 | 103 | 160 |
| over | 0.19 | 0.157542 | 117 | 128 |
| high | 0.12 | 0.236314 | 166 | 89 |
| thicker | 0.19 | 0.144414 | 117 | 138 |
| across | 0.24 | 0.078771 | 99 | 214 |
| base | 0.19 | 0.131285 | 117 | 140 |
| front | 0.19 | 0.131285 | 117 | 147 |
| below | 0.13 | 0.196928 | 153 | 104 |
| end | 0.09 | 0.236314 | 198 | 88 |
| moles | 0.21 | 0.0919 | 111 | 204 |
| which | 0.18 | 0.131285 | 125 | 151 |
| up | 0.13 | 0.183799 | 153 | 117 |
| looking | 0.12 | 0.196928 | 166 | 108 |
| late | 0.11 | 0.210056 | 184 | 102 |
| cropped | 0.09 | 0.223185 | 198 | 94 |
| outer | 0.23 | 0.065643 | 103 | 254 |
| area | 0.18 | 0.118157 | 125 | 153 |
| two | 0.18 | 0.118157 | 125 | 166 |
| both | 0.12 | 0.183799 | 166 | 111 |
| larger | 0.11 | 0.196928 | 184 | 107 |
| near | 0.09 | 0.210056 | 198 | 103 |
| shaven | 0.07 | 0.236314 | 239 | 91 |
| edges | 0.24 | 0.039386 | 99 | 322 |
| cheekbones | 0.14 | 0.144414 | 143 | 132 |
| clean | 0.05 | 0.249442 | 304 | 83 |
| brows | 0.04 | 0.262571 | 363 | 77 |
| even | 0.19 | 0.078771 | 117 | 221 |
| coloured | 0.17 | 0.105028 | 132 | 172 |
| sparse | 0.13 | 0.144414 | 153 | 137 |
| just | 0.12 | 0.157542 | 166 | 127 |
| it | 0.19 | 0.065643 | 117 | 252 |
| be | 0.17 | 0.0919 | 132 | 193 |
| bow | 0.15 | 0.105028 | 137 | 170 |
| faint | 0.12 | 0.144414 | 166 | 135 |
| sized | 0.11 | 0.157542 | 184 | 130 |
| almond | 0.08 | 0.183799 | 216 | 110 |
| eyelids | 0.17 | 0.078771 | 132 | 222 |
| young | 0.17 | 0.078771 | 132 | 235 |
| bulbous | 0.14 | 0.105028 | 143 | 171 |
| hooded | 0.13 | 0.118157 | 153 | 158 |
| peak | 0.13 | 0.118157 | 153 | 161 |
| bit | 0.12 | 0.131285 | 166 | 141 |
| gap | 0.12 | 0.131285 | 166 | 148 |
| shaved | 0.20 | 0.026257 | 115 | 480 |
| thinner | 0.19 | 0.039386 | 117 | 365 |
| an | 0.17 | 0.065643 | 132 | 236 |
| early | 0.14 | 0.0919 | 143 | 196 |
| have | 0.14 | 0.0919 | 143 | 200 |
| little | 0.13 | 0.105028 | 153 | 181 |
| pointed | 0.13 | 0.105028 | 153 | 183 |
| possible | 0.12 | 0.118157 | 166 | 162 |
| wrinkles | 0.12 | 0.118157 | 166 | 168 |
| 20s | 0.11 | 0.131285 | 184 | 139 |
| hazel | 0.09 | 0.144414 | 198 | 136 |
| darker | 0.08 | 0.157542 | 216 | 123 |
| pointy | 0.18 | 0.039386 | 125 | 343 |
| size | 0.18 | 0.039386 | 125 | 351 |
| fairly | 0.15 | 0.065643 | 137 | 250 |
| angular | 0.14 | 0.078771 | 143 | 215 |
| possibly | 0.14 | 0.078771 | 143 | 229 |
| nostril | 0.13 | 0.0919 | 153 | 205 |
| freckle | 0.11 | 0.118157 | 184 | 156 |
| heavy | 0.11 | 0.118157 | 184 | 157 |
| corner | 0.09 | 0.131285 | 198 | 144 |
| edge | 0.09 | 0.131285 | 198 | 146 |
| fine | 0.07 | 0.157542 | 239 | 126 |
| eyelid | 0.15 | 0.052514 | 137 | 278 |
| rectangular | 0.13 | 0.078771 | 153 | 230 |
| burns | 0.06 | 0.157542 | 263 | 121 |
| eyebrow | 0.06 | 0.157542 | 263 | 124 |
| defined | 0.18 | 0.013129 | 125 | 591 |
| blemishes | 0.14 | 0.052514 | 143 | 271 |
| low | 0.13 | 0.065643 | 153 | 253 |
| upturned | 0.12 | 0.078771 | 166 | 234 |
| all | 0.11 | 0.0919 | 184 | 192 |
| protruding | 0.08 | 0.118157 | 216 | 163 |
| distance | 0.06 | 0.144414 | 263 | 134 |
| few | 0.05 | 0.157542 | 304 | 125 |
| reddish | 0.05 | 0.157542 | 304 | 129 |
| seems | 0.15 | 0.026257 | 137 | 478 |
| crown | 0.13 | 0.052514 | 153 | 274 |
| appears | 0.12 | 0.065643 | 166 | 237 |
| forward | 0.11 | 0.078771 | 184 | 224 |
| feathered | 0.09 | 0.0919 | 198 | 197 |
| fuller | 0.08 | 0.105028 | 216 | 179 |
| style | 0.08 | 0.105028 | 216 | 187 |
| back | 0.04 | 0.157542 | 363 | 120 |
| styled | 0.15 | 0.013129 | 137 | 851 |

**SR Word Cloud**

**
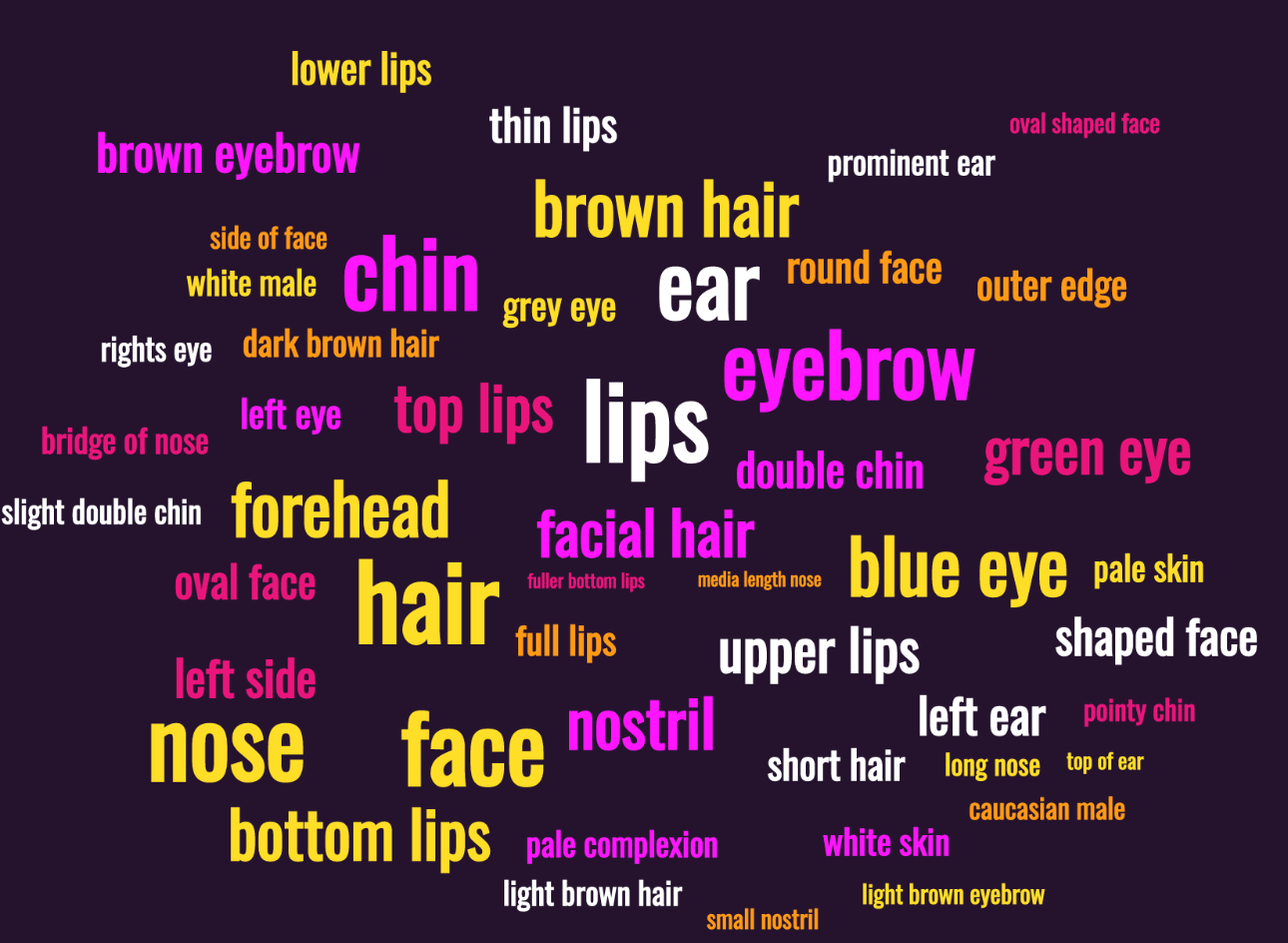
**

**Control Word Cloud**

**
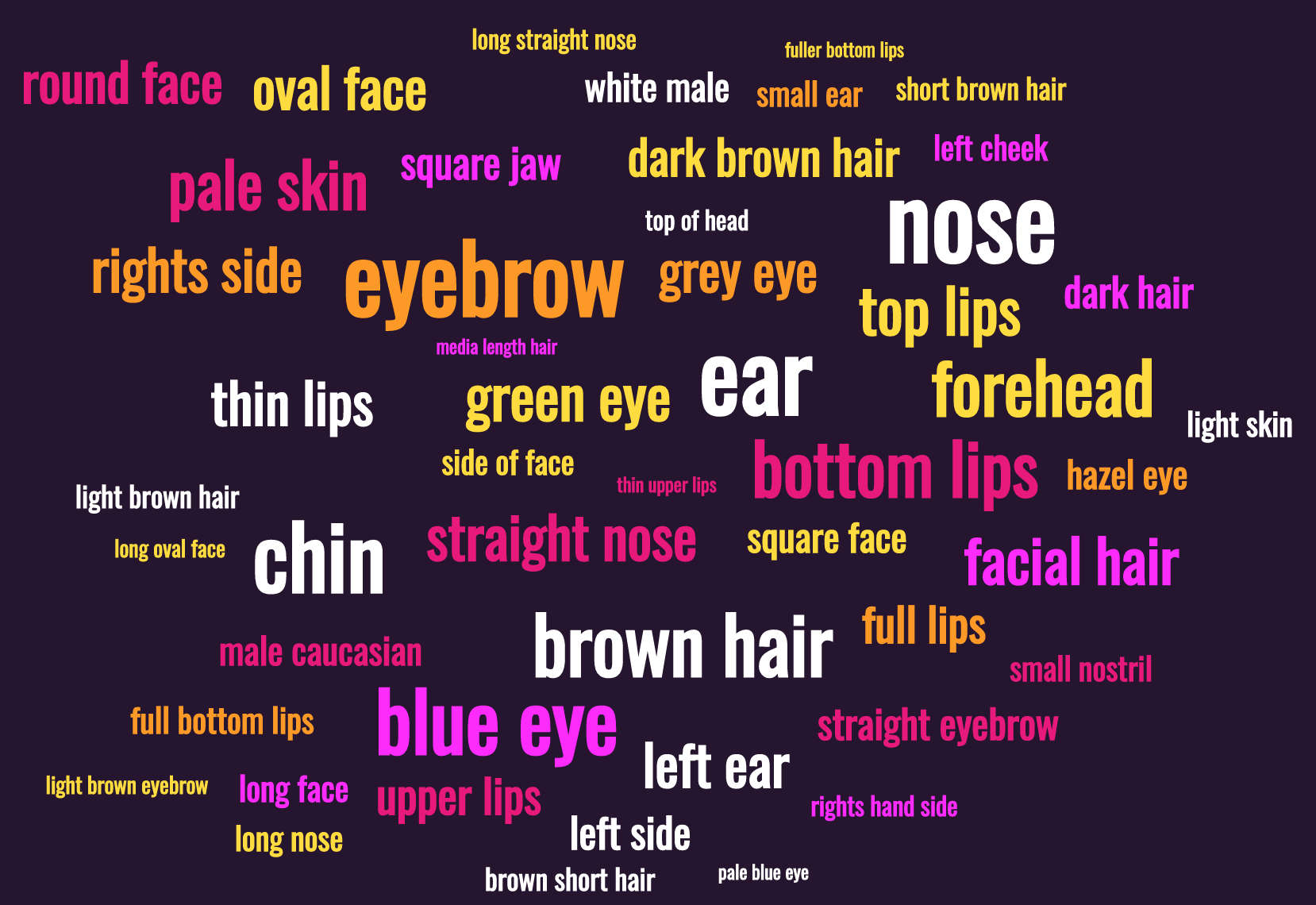
**

**Study 3**

Good and bad descriptions for each trial of Identification-from-Description Task

| **Trial Number** | **Good Description** | **% Correct IDs in Study 2** | **Bad Description** | **% Correct IDs in Study 2** |
| --- | --- | --- | --- | --- |
| 1 | Slightly peroxided hair - straight and messy with strands falling on forehead and sideburn line; looks skunky, slight stubble on upper lip/chin, longer patch of hair directly under lip, pale skin, slightly rosy cheeks, blue eyes. | 0.92857143 | round face, blue eyes, narrow set eyes, narrow nose with bulbous end, think lips, large gap between eyes and hairline, long face, split chin, no prominent cheekbones, dark hair (dyed blonde), blue eyes (darker steel colour), light skin, thin top lip, flat mid sized ears. | 0.4 |
| 1 | young looking male. long straight hair at the top of his head. blonde streaks throughout hair (skunk looking). eyes are closely set to the bridge of the nose. blue coloured eyes. long blonde sideburns. facial stubble with a longer goatee under the bottom lip. | 0.92307692 | sandy coloured dyed hair, mid brown facial hair, pale skin with pink undertones, full bottom lip, thick eyebrows extending beyond inner corner of eyes, double chin | 0.3 |
| 1 | Round face with some brown facial hair along jawline extending to middle of bottom lip. Growth on top lip is angled upwards from outer lip towards nostrils. Light brown medium length hair with longer sections around ears. Blue almond shaped eyes. Full straight brows. Straight nose with a bulbous tip. High fleshy cheeks. Full lips. Cleft chin. | 0.90909091 | brown coloured hair, heavy eyebrows, slight cleft to chin, light growth on face with tuft under lower lip, eyes half-closed, face lopsided to left. | 0.3 |

| **Trial Number** | **Good Description** | **% Correct IDs in Study 2** | **Bad Description** | **% Correct IDs in Study 2** |
| --- | --- | --- | --- | --- |
| 2 | large round narrow set blue eyes (light blue), long nose prominent tip, thin lips, low set eyes, flat ears, round face, no cheek bone prominence, weak jaw and weak chin, long distance between brows and hairline, flat and straight eyebrows, light skin, brown hair, slightly outward sloping eyes, | 0.9 | round deep set blue eyes, dark brows, thin lips, round face, wide flat nose, brown short hair with distinct widows peak, small ears close to head, caucasian | 0.45454545 |
| 2 | male, round face, small double chin, small lips, nose has wide nostrils. blue wide open eyes, straight medium eyebrows, short sideburns. short stubble and a pimple on left cheek and left chin. 2 small moles on right side of upper lip.ears very small and close to head. the left ear is a little higher than the right. hair medium brown and short with a longer fringe in the centre of forehead. | 0.9 | Brown hair, high forehead, brown eyebrows, blue-grey eyes, almond shaped, pale skin, broad oval face and jaw, high cheekbones, rounded chin, normal lips, broad nose | 0.4 |
| 2 | round face, reddish pimple on left side of chin. mole at right-side of lip. mole on left check. scattered freckles on face, black eyebrows, blue eyes. black sideburns, dark hair almost black, fringe at centre of forehead. slight stubble roundish nose | 0.83333333 | chubby dopey looking baby hobbit | 0.09090909 |

| **Trial Number** | **Good Description** | **% Correct IDs in Study 2** | **Bad Description** | **% Correct IDs in Study 2** |
| --- | --- | --- | --- | --- |
| 3 | Brown hair combed into a triangular shape on top of head, looks like a volcano. Fringe has tufts of hair separated along fringe line. Hair comes down to bottom of ears on both sides. Light green eyes , Looking at picture right ear is protruding slightly more than left ear. One freckle just below right eye with another below this one just above right side ear lobe. Also a small blemish adjacent right nostril. Some very minor stubble also appearing. | 0.90909091 | broad eyebrows, big ears dysfunctional nose maybe an accident partial hair growth around the chin | 0.18181818 |
| 3 | bulbous tipped nose very thin in centre. green eyes with large brows. brown hair swept forward medium length. pale in skin colour with a square jawline. | 0.9 | White male, Oval shaped head, short dark hair brushed towards the face with side burns, large downturned eyes the left being slightly lazy. the nose appears to be of thin Greek style full top and bottom lips the top having a slight cupid bow, dark facial hair is obvious if left unshaven. | 0.18181818 |
| 3 | Thick medium brown hair with a spiked chunk at the top and wispy pieces coming down onto the forehead, the hair on the sides of the head above the ears is very thick and rounded, prominent ears that protrude out from the head particularly at the top, hazel eyes with barely visible eyelashes, light natural eyebrows with little hair between them, light brown mole to the left of nose in line with the top of the nostril and edge of left iris, two light brown moles on the upper cheek close to the left ear in line with the outer edge of the left eyebrow, one mole in the stubble low on left of face almost touching jawline, narrow bridge of the nose with an uneven bulbous tip and nostrils, slightly larger than average lips that are evenly proportioned to one another, short dark stubble | 0.81818182 | pale skin, sleepy eye expression, large oval blue/grey eyes, light brown short soft hair, crooked small nose, round face, small mouth | 0 |

| **Trial Number** | **Good Description** | **% Correct IDs in Study 2** | **Bad Description** | **% Correct IDs in Study 2** |
| --- | --- | --- | --- | --- |
| 4 | short side with shag top hair brown in colour with blonde highlights on top. green eyes with darker outer ring. slightly freckled pale skin. rectangular face with square chin. | 0.81818182 | male, oval face with very square jaw and square chin. medium lips, nose has quite blocky looking nostrils - though the nose isn't large. prominent ears that the top of the ear is in line with the eyes. wide grey eyes with prominent darker iris. clean shaven. short cropped light brown hair. small amount of hair on the top a bit longer and almost fuzzy looking with streaks of blonde. | 0.18181818 |
| 4 | short chestnut/blondish hair longer on top with shorter sides. rectangular type face shape. ears protrude slightly. complexion slightly pink orange. no facial hair to speak of. complexion seems clear apart from maybe slight pimpling around the mouth. looks to possibly have a pointy nose with quite narrow nostrils. fairly blank expression with closed mouth. | 0.8 | long narrow face with prominent cheek bones, large flat ears, thin lips, with prominent split(slight) chin, prominent jaw, round hooded eyes, short narrow nose, flat straight eyebrows, light skin, light blue eyes, dark blonde hair, short distance between hairline and brows, | 0 |
| 4 | Blue eyes, possibly green in some light. Short spiked highlighted hair. prominent freckle below right nostril. | 0.8 | Clean cut hair style, Caucasian, roundish face green eyes, little growth around the chin. | 0 |

| **Trial Number** | **Good Description** | **% Correct IDs in Study 2** | **Bad Description** | **% Correct IDs in Study 2** |
| --- | --- | --- | --- | --- |
| 5 | short spiky brown hair that is lighter in parts. Blue eyes, moustache and goatee beard stubble. Square jawline | 0.93333333 | Short slightly messy brown hair, short sideburns, short black stubble across upper lip/most of chin, blue eyes, right eyelid more exposed, mole on his left cheek, head slightly leaning to the left. | 0.18181818 |
| 5 | Round face, blue eyes. Dark medium short hair. Stubble above lip and lower chin. Freckle to left of nose. | 0.92307692 | pale skin with blue eyes, sandy coloured hair with red tinge, stubble on face, thicker eyebrows, full lips bowed in centre, straight proportioned nose, ears have lobes joined to face and a curve two thirds of the way up. left eyebrow could be a slightly different shape to right eyebrow. left eye is more hooded than right | 0.16666667 |
| 5 | hooded narrow set light blue eyes, short narrow nose, thin lips, weak chin, prominent jawline, flat ears, dark hair, flat forehead, round face, outward sloping eyes, slightly prominent cheekbones, light skin | 0.90909091 | White male diamond shape face, brown shaggy short hair, side burns, thickish eyebrows, down turned eyes colour blue, slightly wide nose, mole to left of nose, different shaped ears right ear is top notched and left slightly inverted lower side, thick top and bottom lips , mild facial beard hair. | 0 |

| **Trial Number** | **Good Description** | **% Correct IDs in Study 2** | **Bad Description** | **% Correct IDs in Study 2** |
| --- | --- | --- | --- | --- |
| 6 | hair almost black, straight with slight fringe. clean shaven with black 5 oclock shadow. complexion considerably pale. slight pimpling on left side with one large pink blemish on left side close to nose. Large thick lips with slightly open mouth, a gap between his front teeth. slight darkening under the eyes. green eyes. No expression to speak of, could be described as a blank stare. Oval roundish face. | 1 | male, Caucasian, chubby, oval shaped head, short dark hair, heavy eyebrows, light stubble, ears close to the head, full lips. grey/green eye, one obvious freckle on LHS cheek below mouth line | 0.58333333 |
| 6 | Round head, thick black hair that frames round head, puffy face and lips, pale skin, double chin,empty green eyes, gap between front teeth, handful of pimples around nose. | 1 | - Male - Caucasian - 24 - 28 - No visible wrinkles - Larger, round, oval face with beginnings of a double chin - Does not have a strong jawline or cheekbones - Pasty skin tone, minimal blemishes in t-zone - Full dark brown eyebrows with arch beginning at temple & the eyebrow hair feathered in centre near nose - Round medium length nose - Round full lips - Gap between front top teeth - Dark brown hair with feathered fringe & medium to long sideburns  - 1 day growth for light stubble - Blue green coloured round eyes | 0.5 |
| 6 | fleshy pale skin face, double chin, upturned nose, pale blue eyes, full lips, small close set ears, gap in front teeth, dark brown facial hair | 1 | oval face with dark brown medium length hair swept to one side.thin dark brown brows. greenish blue eyes. triangular nose, skin has acne and quite blotchy. double chin. | 0.5 |

| **Trial Number** | **Good Description** | **% Correct IDs in Study 2** | **Bad Description** | **% Correct IDs in Study 2** |
| --- | --- | --- | --- | --- |
| 7 | chestnut coloured hair. Shaved head to maybe 5mm from scalp. angular oval shaped face. His left ear protrudes more than his right ear. Slight hint of a smile with closed thin lips. friendly unassuming look. nose seems to start centrally but angles to his left side. pale complexion with no noticeable blemishes. eyebrows seems to maybe be slightly more blonde than his head hair. | 0.9 | Brown hair crew cut, pale brown eyebrows, dark blue almond shaped eyes, straight nose bridge, square face, slightly rounded jaw, pale skin, normal cheekbones, thin lips | 0.2 |
| 7 | Crew cut hair style, rounded face, ears stand out | 0.9 | short cropped shaved head in a pale brown blonde colour matching eyebrows thicker towards the centre. prominent eyes. Chin is quite square with very slight dimple. more rectangular face | 0.2 |
| 7 | Symmetric face, left ear wider Large open relaxed eyes Short cropped light brown hair Late 20's Large protruding chin with small dimple Small, thin bottom lip Pale skin Possible scar to centre hairline Wide forehead | 0.9 | oval shaped head with a more squoval shaped chin. right eye (when viewing face) sticks out further than the left. mousey brown hair colour. very short hair (1 or 2). rounder eye shape with blue/greyish eye colour. facial stubble mousey brown colour. | 0.125 |

| **Trial Number** | **Good Description** | **% Correct IDs in Study 2** | **Bad Description** | **% Correct IDs in Study 2** |
| --- | --- | --- | --- | --- |
| 8 | sharp v shaped chin, triangular nose, small blue eyes, ginger hair | 0.9 | Caucasian male with fair-pink skin and green or blue eyes. Short, thick, red hair that would possibly be curly if allowed to grow. High forehead, small well defined features, eyes are slightly close set. Straight nose, thin bridge, rounded tip. Thinner upper lip, full lower lip, overall mouth is small. Oval face shape with a softer jawline, well defined chin. Sparse facial hair, also red. Right ear sticks out slightly more than left ear. | 0.25 |
| 8 | Short red hair that's slightly wavy, pointy jaw, pointy right ear, slight re stubble on jawline and upper lip, small round lips, tired green eyes. | 0.81818182 | red facial hair, blue eyes, pale skin, ears stick out slightly, no earlobes, left eyebrow is missing hair due to fading scar on left side of face, straight upturned nose, thin upper lip | 0.23529412 |
| 8 | The subject is white/Caucasian and a redhead with a ruddy-complexion consistent with red hair. The face shape is oval with a pointy chin. The hair is 2-3cm in length and looks styled with product into a groomed neat style. The ears are small and elfin, pointy at the top descending to very small or non-existent lobes. The eyes are small, sharp and blue-green-to-grey. The eyebrows are red with a scar at the top of his left eyebrow. The eyebrows are thinner at the bridge of the nose and fan out at the outer edges. The nose is straight and medium sized without any prominent deformations. The mouth is small and bow shaped. The top lip is especially small and the bottom lip is rounded and neat. The subject has reddish short stubble, quite scanty especially on the upper lip. The subject has some forehead wrinkles and looks mid-to-late 20's. | 0.8 | Red hair and stubble, normal length hair, red eyebrows, blue-green eyes, wide bridged nose, non-prominent nostrils, pinned-back ears, very pale skin, full lips, centre-weighted, rectangular face, short jaw, squared-off chin | 0.2 |

| **Trial Number** | **Good Description** | **% Correct IDs in Study 2** | **Bad Description** | **% Correct IDs in Study 2** |
| --- | --- | --- | --- | --- |
| 9 | light coloured freckly skin, brown hair long around face, brown stubble, blue thin downturned almond shaped eyes, thin oval shaped face, light brown eyebrows, big long nose, thin upper lip, features in proportion with the rest of face | 1 | blue eyes with pale freckled skin. medium ginger brown beard. quite a wide nose in the mid section. dark ginger brown shag hair. upper eyelids are quite low and hang over top lid. brows are same ginger brown as growth on face. | 0.6 |
| 9 | young male (25-30). long brown hair covers ears, sweeps across forehead towards one side of his face. freckles across nose. pointy chin. round bridge of nose. stubble around cheeks, chin and upper lip. blue eyes with an almond shape that slightly hangs downwards. | 0.90909091 | straight moppy dark brown hair. brown facial hair with moderately thinner hair around the moustache. Stern or forlorn expression with blue eyes. Pale complexion with an odd couple of freckle blemishes between eyes/top of the nose. almost pursed lips with defined cupids bow on upper lip. | 0.33333333 |
| 9 | white male, Dark Brown collar length hair possible dyed fringed to the right, Diamond shaped head, down turned Blue eyes, lightly freckled Nubian nose, strong Cupid Bow top lip and full lower lip and reddish tinge short facial hair. | 0.90909091 | long narrow nose, thin top lip, long face with prominent high set cheekbones, large eyes, regular set distance eyes, prominent large ears, flat and long brows, light skin, brown hair | 0.3125 |

| **Trial Number** | **Good Description** | **% Correct IDs in Study 2** | **Bad Description** | **% Correct IDs in Study 2** |
| --- | --- | --- | --- | --- |
| 10 | short sandy coloured hair, straight full eyebrows, pale blue eyes, thin lips, straight nose with small nostrils, oval shaped face, ears have dents at the top where they curve | 1 | heavy hooded (small distance between brows and upper eyelids) light blue wide set eyes, long narrow nose, prominent ears (large), long narrow face, prominent jaw and chin, long distance between nose and top lip, thin lips, short distance between hairline and brows, no prominent cheekbones, dark blonde hair and slightly tanned skin. | 0.45454545 |
| 10 | Male, youngish. Long face, thin lips, long nose, long chin. Blueish eyes, narrow in photo, no visible upper eyelid. Kink in ears near the top. ears obvious as hair is cropped very close. Widow's peak. Brown hair. Straight eyebrows. Sparse moustache, sparse hair around chin. | 1 | long round face, ears have folds almost at top. light brown hair, similar to army style hair cut. no fringe, thinning out on sides, full exposure of forehead. eyebrows are dark and thinnish. eyes are pale greyish, squinting. no blemishes or freckles. thin pale pink lips. stubble on chin, possibly 2 days old worth. less stubble coming from both nostrils then going around lips. | 0.45454545 |
| 10 | squinty blue eyes, thin mouth, light facial hair and crew cut | 0.90909091 | Dark brown hair, crew cut, bushy brown eyebrows, blue-grey narrow eyes, broad nosebridge, non-prominent nostrils, round-profile ears, thin lips, brown stubble, long rectangular face, long jaw, squared-off chin | 0.45454545 |
